# *Xtricorder*: a likelihood-enhanced self-rotation function and application to a machine learning-enhanced Matthews prediction of asymmetric unit copy number

**DOI:** 10.1107/S2059798325009647

**Published:** 2025-11-26

**Authors:** Airlie J. McCoy, Randy J. Read

**Affiliations:** a Cambridge Institute for Medical Research, Department of Haematology University of Cambridge The Keith Peters Building, Hills Road CambridgeCB2 0XY United Kingdom; European Bioinformatics Institute, United Kingdom

**Keywords:** molecular replacement, Matthews coefficient, machine learning, *Phaser*, *Phasertng*, self-rotation function

## Abstract

Analysis of crystallographic diffraction data after collection and integration but before phasing gives the crystallographer a ‘first-look’ assessment of data quality and flags potential challenges in subsequent structure determination. We here report the development of *Xtricorder*, a ‘first-look’ application specifically targeted at likelihood-based phasing. *Xtricorder* incorporates the full array of analyses previously available in the *Phaser* codebase, with some enhancements and updates, in a more streamlined and accessible implementation. In addition, *Xtricorder* offers a likelihood-enhanced self-rotation function. A novel graphical representation of the self-rotation function, the ‘composite-section diagram’, presents the results for user inspection and has the added advantage that, in an adapted form, it is appropriate for training a convolutional neural network to enhance the standard Matthews analysis and double the accuracy of asymmetric unit copy-number prediction. We investigate the usefulness of the likelihood-enhanced self-rotation function in ‘first-look’ analyses, exploring the circumstances under which the self-rotation function results are useful, and discuss the application to AI-generated structure prediction.

## Abstract 2

*Xtricorder* is a tool for analysing crystallographic data, featuring a likelihood-enhanced self-rotation function and a novel ‘composite-section diagram’ thereof, which aids interpretation and enables a machine learning-enhanced prediction of asymmetric unit content.

## 1. Introduction

*Xtricorder* for the analysis of integrated crystallographic data builds on software already available that has been developed both by our group and others. Indeed, the name is partly inspired by the name of *Xtriage* (Zwart *et al.*, 2005 ▸), a component of the *Phenix* suite (Liebschner *et al.*, 2019 ▸) which performs diagnostic tests for crystallographic structure solution. *Xtriage* assesses the overall completeness, redundancy and the presence of possible pathologies such as anisotropy, translational noncrystallographic symmetry (tNCS), twinning and pseudo-symmetries, and has been extensively cited as a useful ‘first-look’ tool.

The *CCP*4 suite (Agirre *et al.*, 2023 ▸; Winn *et al.*, 2011 ▸) has programs that fulfil a similar role, although they are not wrapped in a single application: *CTRUNCATE* for anisotropy analysis and twinning tests (French & Wilson, 1978 ▸), *POINTLESS* for space-group analysis (Evans, 2006 ▸), *Zanuda* for space-group subgroup analysis (Lebedev & Isupov, 2014 ▸) and *AUSPEX* for visual inspection of data-intensity pathologies (Thorn *et al.*, 2017 ▸). In addition, *CCP*4 has two implementations of a self-rotation function: one in *MOLREP* (Vagin & Teplyakov, 2010 ▸) and one in *POLARRFN* (Agirre *et al.*, 2023 ▸; Winn *et al.*, 2011 ▸).

Our software *Phaser* (McCoy *et al.*, 2007 ▸), distributed with both *Phenix* and *CCP*4, performs an array of analyses that are necessary/optimal for application of the maximum-likelihood functions for phasing by molecular replacement (MR), single-wavelength anomalous dispersion (SAD) and the combined MR-SAD method. These also offer useful ‘first-look’ analyses: correction of systematic intensity modulations due to anisotropy and translational noncrystallographic symmetry (tNCS; Caballero *et al.*, 2021 ▸; Read *et al.*, 2013 ▸), cell-content analysis to estimate the fraction of scattering atoms/models for σ_A_ estimation (Read, 1986 ▸), and twinning tests (Padilla & Yeates, 2003 ▸), particularly in the presence of tNCS. The presence of twinning modifies the target for the expected log-likelihood gain-directed optimization of the resolution for phasing (Oeffner *et al.*, 2018 ▸). Additionally, reflections are analysed for information content and the set of reflections is filtered to remove those reflections that only slow down the calculations.

The reorganization, modifications and expansion of the codebase on moving from *Phaser* to *Phasertng* (McCoy *et al.*, 2021 ▸) offered the opportunity to not only make this functionality available in a dedicated standalone application but also offered the opportunity to add a self-rotation function (SRF). By wrapping these functionalities, we aim to make them more accessible and visible to crystallographers, so as to encourage uptake.

The SRF provides insight into rotational relationships within the crystal lattice. Historically, it has been used on an *ad hoc* basis to discern the point-group symmetry of oligomers and indications of the number of copies in the asymmetric unit (Rossmann & Blow, 1962 ▸). However, the SRF is not currently standardly applied in structure-solution pipelines, nor is it automatically mined for information pertinent to structure solution *a priori*. We envisage *Xtricorder* being added to structure-solution pipelines at the step between integration and phasing. The source of integrated data, whether from traditional oscillation images integrated with, for example, *XDS* (Kabsch, 2010 ▸), from serial crystallography at XFEL sources integrated with, for example, *Cheetah*/*CrystFEL* (Barty *et al.*, 2014 ▸; White *et al.*, 2012 ▸) or from serial crystallography at synchrotrons, integrated with, for example, *DIALS* (Beilsten-Edmands *et al.*, 2024 ▸), is not an issue in the analysis.

Any internal symmetry of an oligomer is known to be an advantage during crystallization (Chruszcz *et al.*, 2008 ▸) and has even been proposed as a mechanism to improve crystallizability (Banatao *et al.*, 2006 ▸). Many proteins form oligomers in solution with a point-group symmetry that is capable of being represented by a crystal symmetry (for example *C*_3_ trimers in *P*3, *D*_2_ tetramers in *P*222); however, during crystallization these point groups are not always incorporated as crystallographic symmetry. Likewise, an oligomeric point-group symmetry for which a subgroup of the point group may be represented by crystal symmetry (for example *C*_12_ in *P*6) may or may not be partly crystallographic. Conversely, proteins that are not oligomeric in solution may crystallize with noncrystallographic symmetry (as well as crystallographic symmetry). Therefore, even when the SRF gives a clear signal for symmetry, the relationship between the SRF-identified symmetry and the oligomeric associations and asymmetric unit composition is far from obvious or straightforward. Biophysical analysis of the oligomerization state is invaluable in this regard.

In the era of AI-generated structure prediction, the SRF also has the potential to provide useful information about oligomeric associations for structure prediction. In the absence of a structure, determination of the oligomeric state can direct structure-prediction protocols to target a particular oligomeric state and thereby improve predictions, which may tip the scales in improving the model for molecular-replacement phasing. It is therefore of interest to look again at the information that can be provided by the SRF in a systematic study.

## 2. Self-rotation function

The SRF identifies rotational relationships in the crystal, including symmetries, by comparison of the data against themselves. Only point-group symmetry is detected in this analysis: any translations associated with the rotations are not identified (for example, 2_1_ screw rotations are reduced to twofold rotations). Crystallographic and noncrystallographic point groups are described in Section 10.1 of Volume A of *International Tables for Crystallography* (Hahn & Klapper, 2006 ▸).

SRFs have long been implemented in the formerly popular programs *X-PLOR* (Brünger, 1992 ▸) and *CNS* (Brunger, 2007 ▸), and in the currently popular programs *MOLREP* and *POLARRFN*. These implementations use the Patterson function, which is the Fourier transform of the intensities and which gives a vector map of the crystal (for a review, see Evans & McCoy, 2008 ▸). Vectors close to the origin represent intramolecular associations and associations at crystal contacts, while at increasing distances from the origin increasing proportions of vectors represent intermolecular relationships. Cutting out a sphere around the Patterson origin enriches the Patterson for intramolecular vectors.

Since rotations of molecules are echoed in the rotations of the corresponding vectors, the molecular rotations are identified by a match of the radius-restricted Patterson against a copy of itself after rotation. The match can be measured as various functions, such as a product function or a correlation coefficient, or as the equivalent to the Patterson product function in reciprocal space. The reciprocal-space version can be made fast by factorization and fast Fourier transform (FFT), and is known as the ‘fast rotation function’ (Crowther, 1972 ▸; Navaza, 1994 ▸). The highest peak is at 0° rotation, and the height of other peaks is normally measured as the percentage of this (100%) ‘origin’ peak, with the mean taken as 0%. In this way, the SRF is analysed analogously to the way that the native Patterson is used to identify tNCS, where non-origin peaks representing molecular translations are identified by their height relative to the (100%) ‘origin’ peak which corresponds to the molecule mapped to itself (Caballero *et al.*, 2021 ▸).

## 3. Likelihood-enhanced self-rotation function

The established fast rotation function in *Phaser* is based on the likelihood-enhanced rotation function (LERF; Storoni *et al.*, 2004 ▸). First-order and second-order approximations of the full likelihood rotation function were developed as LERF1 and LERF2. LERF1 was found to be faster (requiring only one FFT rather than two) and sufficient to find the correct orientation, and LERF2 was consigned to developer use only. Only LERF1 has been ported to *Phasertng*.

In the context of the rotation function of model against data used for molecular replacement (the cross-rotation function; CRF), LERF1 has the major advantage over the Patterson rotation function of being able to incorporate information from a fixed partial model and hence substantially enhance the signal for second and subsequent asymmetric unit model components when the asymmetric unit is built up by addition, a hallmark of the maximum-likelihood approach. Where there is no fixed model, and as originally published, LERF1 for the CRF reduces to the fast rotation function proposed by Bricogne (1997 ▸). The coefficients for the data component are 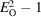 , where *E*_O_ is the normalized observed structure-factor amplitude, while the coefficients for the model component are 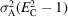, where *E*_C_ is the normalized calculated structure-factor amplitude (from the model) and σ_A_ is the estimated correlation between the true and calculated structure-factor amplitudes. These coefficients correspond to sharpened, variance-weighted, origin-removed Pattersons.

Since the publication of the LERF, we have developed the log-likelihood gain on intensity (LLGI) target for molecular replacement, which has improved the coefficients for LERF1; LLGI-LERF1 has *E*_O_ replaced by *D*_obs_*E*_eff_ (Read & McCoy, 2016 ▸). These coefficients incorporate the error in the observed intensity into the likelihood target through a transformation that preserves the correct normalization of the data, particularly for data with low *I*/σ(*I*), avoiding the distortions introduced using the ‘inflation of the variance’ method (for a review, see McCoy, 2004 ▸). For the likelihood-enhanced SRF (LESRF), the coefficients are 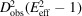. The LESRF is highly analogous to the LERF1 (CRF) and no further computational tools are required.

An advantage of using the LESRF over the Patterson-based SRF is a reduced dependency on the resolution of the data, because data with low *I*/σ(*I*) are intrinsically downweighted through low values of *D*_obs_. The LESRF also includes the anisotropy correction and tNCS correction terms in the calculation of *E*_eff_.

## 4. Graphical representations

The fast SRF (like the fast CRF) results in a three-dimensional map calculated in Euler angle space, with peaks at the positions of both the crystallographic and noncrystallographic rotations. *Xtricorder* follows the tradition of other SRF software by projecting the contours for visualization using polar (stereographic) plots. The Euler angles of the calculation space are refactored as axis (φ and ψ) and angle (χ) rotations, with a polar plot for each angular χ section. The contour plot for each χ section (range 0–180°, normally at intervals of between 1° and 5°) allows the user to identify peaks that represent the direction of the axis of the rotation, if any. In *Xtricorder*, the SRF has a minimal sampling of 3° independent of resolution or molecular radius (unlike the implementation of the CRF) to always to sample χ sections associated with *C*_*n*_ rotations, such as twofold, threefolds and fourfolds, effectively.

### 4.1. Composite-section diagram

The SRF in *Xtricorder* is reported as a novel graphical representation: the ‘composite-section diagram’. This diagram facilitates the identification of significant rotations and their directions and reveals the spatial patterns of crystallographic and noncrystallographic symmetry. The diagram marks the positions of peaks corresponding to rotations of 360°/*n* for *n* up to 24 (*i.e.* composite for χ in the range 15–360°). Marker colour, shape and size are used to indicate SRF peak properties.

Marker size (area) is used to indicate the peak height relative to the original peaks, where the top peak is 100% and the mean is 0%. Crystallographic symmetry operations will generate peak heights near 100% of the origin peak, as will very strong noncrystallographic symmetries.

Marker colour is used to distinguish the nearest integer *n* for the χ rotation of each peak described as 360°/*n* and *n* in the range 2–12: red (2), gold (3), orange (4), light green (5), forest green (6), cyan (7), blue (8), plum (9), magenta (10), burlywood (11) and brown (12), where colours are as defined in *matplotlib* (Hunter, 2007 ▸).

Marker shape is used to distinguish between the ‘exactness’ of the rotational symmetry to a proper rotation. Crystallo­graphic symmetry (*n* = 2, 3, 4 or 6) is marked with stars. Noncrystallographic rotational symmetries 360°/*n* for *n* in the range 2–12 are marked with circles if *n* is an integer (with a small tolerance) and a hollow triangle if outside the tolerances.

Higher order rotations 360°/*n* for *n* ≥ 13 are uncommon. The ability to distinguish the precise order *n* of the rotation becomes increasingly difficult as *n* increases; real variation in the orientation of monomers within higher order multimers in the crystal can degrade the signal. These χ rotations are also less likely to correspond to *C*_*n*_ symmetry. Consequently, rotations 360°/*n* for *n* in the range 13–24 are all displayed with a black cross. Peaks corresponding to *n* > 24 (χ < 15°) are not displayed. Details of these higher order peaks are reported in the logfiles.

The default initial threshold peak height for plotting is 5% of the maximum (origin) peak, which may select many peaks for display. To reduce noise, a secondary, stricter selection is applied after rescaling the peak heights so that the top non­crystallographic (NCS) peak, rather than the origin peak, is treated as 100%. Note that after rescaling, in cases of weak NCS the origin peak can be much higher than 100%, sometimes exceeding 1000%, whereas in cases of strong NCS the origin peak remains close to 100%. Peaks below 30% of the rescaled maximum are then discarded. This means that for strong NCS, the effective minimal peak height relative to the origin is roughly 30% rather than 5%, while for weaker NCS the effective threshold relative to the origin falls between 5% and 30%, allowing significant NCS peaks to be emphasized while suppressing noise.

**Figure 1:**
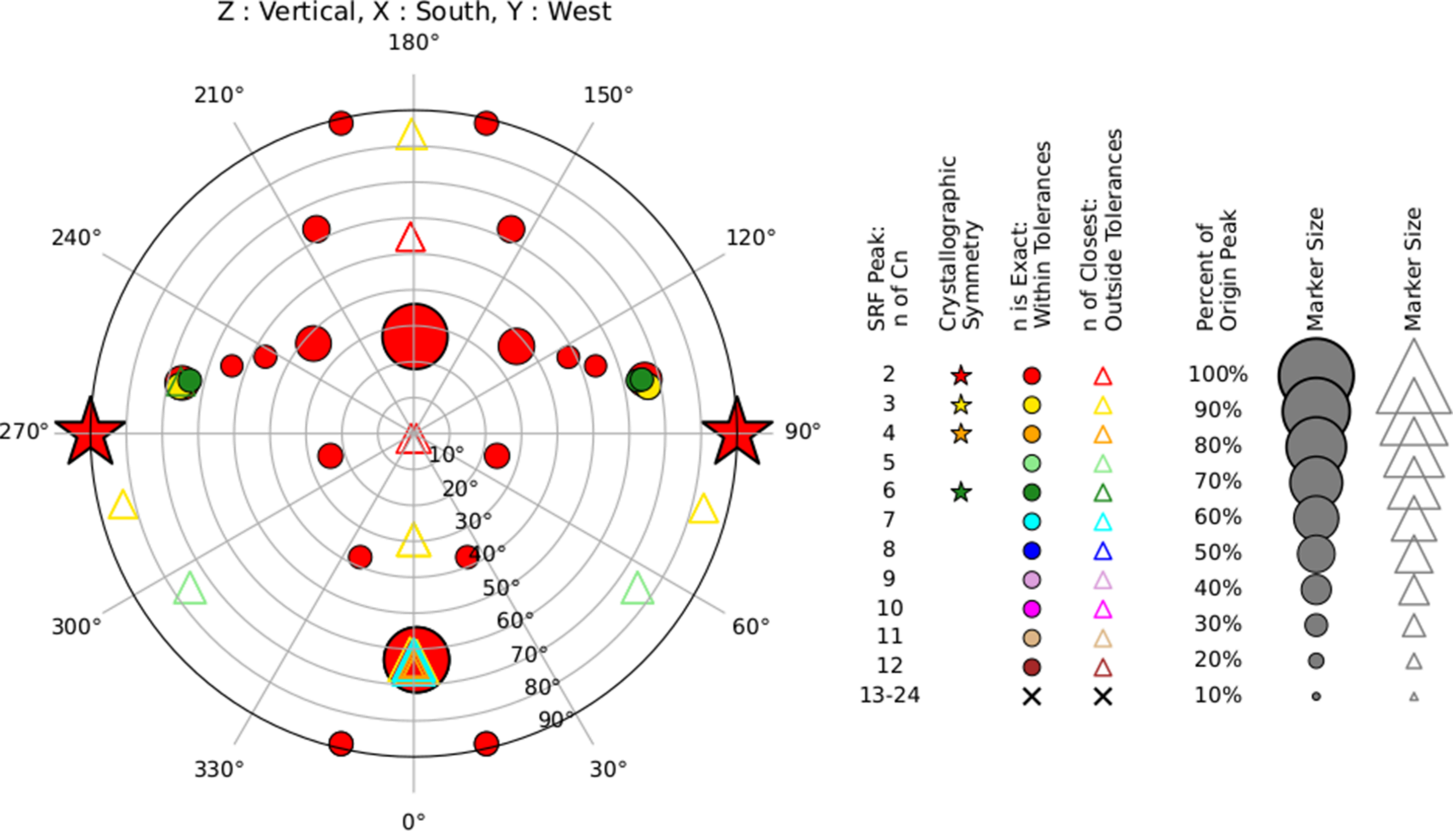
Composite-section diagram for PDB entry 1zye showing a stereographic projection of the SRF peak positions. See the text for details.

The composite-section diagrams are displayed as stereographic and Mercator plots. An example plot for a two-ring catenane comprising two interlocking dodecameric toroids deposited in the PDB as entry 1zye (Cao & Isaacs, 2007 ▸; Supplementary Fig. S1) is shown in Fig. 1 ▸.

### 4.2. Contour plots

The SRF in *Xtricorder* is also rendered as traditional contour plots, offering a complementary perspective that emphasizes the sharpness/shallowness of the SRF peaks selected for the composite-section diagram. The composite-section diagram may be superimposed onto these contours, with peaks corresponding to different *C_n_* symmetries separated onto their corresponding χ section. This view, with the composite-section diagram markers distributed to different layers, gives clarity where there are overlapping markers in the composite-section diagram and ensures that the plots remain interpretable to crystallographers with colour-vision deficiencies.

Cross-referencing the contour plots with the overlaid composite-section diagram enables a more nuanced interpretation of peak features, allowing crystallographers to distinguish between genuinely discrete rotational peaks and shoulders or extensions of broader features.

The contour plots for selected layers of PDB entry 1zye are shown in Supplementary Fig. S3.

### 4.3. Machine-learning representations

The composite-section diagram designed for user interpretation of the SRF results was adapted for machine learning. Constant elements of the plot (the axes and axes labels and annotations) were removed, and the resolution was lowered to 112 × 112 pixels. This size is one quarter of the widely adopted size in machine learning for image classification of 224 × 224 pixels, as the features of the SRF plots are sparse. This size balances preserving sufficient detail while keeping the final weights file small. Rather than using colour to distinguish rotational order on one image, the rotational symmetries *C*_2_–*C*_12_ were output on 11 different images, to separate markers that overlap on the composite-section diagram. Circles and triangles indicated the relationship of the rotation to circular symmetry *C*_2_–*C*_12_ as above; however, the low resolution meant that greyscale was also used to distinguish the two: circles in black and triangles in grey. Size indicated peak height. Rotational symmetry over *C*_12_ was output on image 12 with inverted triangles (*C*_13_), squares (*C*_14_), pentagons (*C*_15_), hexagons (*C*_16_) and crosses (*C*_17_–*C*_24_).

In addition to the 12 SRF images, an additional image, the ‘Matthews-data’ image, encoded the Matthews coefficient probabilities and space group. The probabilities were output in the form of a greyscale heat map, with the intensity of the marker at each position indicating the probability. The space group was encoded as a shape with numbers for the space-group point group superimposed. Information about the most prominent circular (*C*_*n*_) symmetry and any dihedral (*D*_*n*_), octahedral (*O*_t_) and icosahedral (*I*_h_) symmetries were also encoded on this image.

The machine-learning images for PDB entry 1zye are shown in Supplementary Fig. S4.

## 5. Data-set generation

To test the properties of the LESRF, a data set of structures with and without multiple copies in the asymmetric unit was generated from the PDB archive and probed to understand the dependency of the SRF on the radius of integration and the relationship between SRF-identified rotational symmetries and asymmetric unit contents.

### 5.1. Sampling of asymmetric unit copy number and space groups

A search of the PDB was performed to identify structures with up to 24 protein monomers or heterodimers (referred to henceforth as the ‘assembly’) in the asymmetric unit. The number of cases counted decreased rapidly with increasing numbers in the asymmetric unit, and even numbers were more prevalent than odd numbers. Previous analysis of the PDB revealed the same trends (Berman *et al.*, 2013 ▸). A separate search was performed for the set of space groups in each of the 11 point groups, to evenly sample space-group point groups (SGPGs). A limit of 100 test cases per SGPG prevented the vast overrepresentation of some asymmetric unit/space-group point group combinations.

Additional constraints on the selection of test cases were that they had data deposited, *R* factors that were reproducible with *PDB-REDO* (Joosten *et al.*, 2012 ▸) and a resolution of diffraction of at least 3 Å. Likewise, the search was performed separately for proteins under 100 amino acids and over 100 amino acids to ensure a range of molecular sizes. Structures with DNA and RNA were excluded as these have strong internal rotational symmetries. Structures consisting of only coiled coils with a length-to-breadth ratio over 8 were excluded, also because of the strong internal twofold symmetry. Viruses (capsids) were excluded as these are special cases, often having extremely high solvent content, and often having icosahedral symmetry, which is highly unlikely to be unknown to the crystallographer before crystallographic investigation. The search returned representative structures with a sequence-similarity cutoff of 90%.

Data and structures for each identified PDB entry were downloaded from *PDB-REDO*, and the coordinates of one assembly (for example, chain *A* in the case of monomers) were extracted to a separate PDB file. Various annotations associated with the PDB entry were extracted to a database: from the PDB archive came the title, the space group, the resolution of the diffraction data, the number of chains, the number of protein entities and the number of residues, from the PDB-REDO databank came the presence of twinning (as recorded in the PDB header after *PDB-REDO* refinement) and from *Xtricorder* came the solvent content for the deposited structure, the data anisotropy measured as Δ*B* in intensities between strongest and weakest diffraction directions and the model sphericity measured as the ratio of the largest to the shortest extent of the assembly.

The number of cases of seven, nine and 11 in the asymmetric unit were supplemented for the machine-learning studies by applying allowed re-indexing operations to the data, which had the effect of changing the representation of the SRF peak positions (there were only minor computational differences). In addition, five structures with seven in the asymmetric unit for which experimental data were not available were included by using calculated data.

Histograms showing the distribution of properties in the database are shown in Supplementary Fig. S5.

### 5.2. Data-set extension for *P*1 low copy numbers

The data set above was expanded for the special cases of one or two assemblies in the asymmetric unit and space group *P*1 to give more data to compare the LESRF from experimental and calculated data. A further condition was added to test-case selection for two assemblies in the asymmetric unit: the root-mean-square deviation for all atoms between the two copies of the assembly was restricted to less than 1 Å. We refer to the resulting data set of *P*1 with one copy in the asymmetric unit as the ‘P1-monomer’ data set and that with two copies in the asymmetric unit as the ‘P1-dimer’ data set.

To generate calculated structure factors for the assembly suitable for a SRF, another protocol from the *Phasertng* suite, *Nomad*, was used. Structure factors were calculated for a *P*1 box with cell dimensions twice the monomer/dimer molecular radius (note that these calculated structure factors do not use the unit cell of the crystal). The large unit cell ensures that the radius of integration contains only intramolecular vectors. The calculated structure factors could be used in place of experimental data in *Xtricorder* by ignoring the phase component, reading the σ_A_ values as *D*_obs_ and the *E*_C_ values as *E*_eff_.

## 6. Radius of integration

The SRF (Patterson and LESRF) has a major dependency on the integration radius. If the radius is too small, the function will emphasize secondary-structure features such as the symmetry of parallel helices, whereas a radius that is too large will introduce noise from intramolecular vectors.

How well the intramolecular vectors are isolated from the intermolecular vectors by restriction of the radius of integration depends on the sphericity/anisotropy of the atomic distribution of the biological assembly (the macromolecular monomer or macromolecular complex) and the packing/assembly associations in the crystal (Evans & McCoy, 2008 ▸). Although the former may be estimated prior to structure solution (see below), the latter cannot.

The integration radius for the SRF is maximally twice the molecular radius, where all vectors are included, but there is no consensus on the appropriate fraction/multiple (less than 2) to use to optimize the signal in the SRF.

### 6.1. Estimation of the molecular radius

In *Xtricorder* the user has three options for providing information about the molecular radius.

If a model is provided, then the molecular radius is taken as the radius that encompasses 90% of the atoms in the model, *R*_M90_. By taking the radius encompassing 90% of the atoms, volume added by extended N- and C-termini and other low-density/not-compact components of structure at the surface is excluded, since this will not contribute significantly to the Patterson density.

Alternatively, if the molecular weight is provided (or the sequence is provided, from which the molecular weight can be calculated) then the molecular radius is estimated as
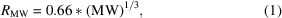
where *R*_MW_ is the minimum radius in ångstroms that can encompass molecular weight MW in daltons (Erickson, 2009 ▸).

Lastly, the user may input a molecular radius explicitly (*R*_i_). The fallback is a default molecular radius of 18 Å, which corresponds to a MW of 30 kDa via equation (1), with 30 kDa being the average molecular weight of a protein domain, which is likely to be the smallest component of any assembly crystallized.

The model-, sequence- or user-derived estimates of the molecular radii (*R*_MW_, *R*_M90_ or *R*_i_) are then adjusted by a scale factor (*k*) of maximum value 2 to give the SRF radius of integration (*b*). Optimization of *k* is discussed below.

Note that *Xtricorder* does not require any information about the assembly radius/model/sequence for the data-analysis steps, although it is used for the cell-content analysis step if provided.

### 6.2. Constraint on radius of integration

There is a limit on the computational stability of the SRF, with the function being unstable beyond a spherical Bessel function *L*_max_ of 100. The *L*_max_ of the spherical Bessel functions in turn depends on the radius of integration (*b*) and the maximum resolution (*d*_min_) of the data through the formula
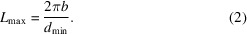
There is thus a limit on the radial resolution of the analysis, either through the data resolution or maximal vector length, the latter because the maximal vector length determines the sensitivity of a change in atomic positions (at the vector-defined molecular boundary) to a given rotation angle.

In *Xtricorder*, the default resolution for the SRF is 3 Å, so the maximal integration radius (*b*_max_) is 48 Å. Using an integration radius equal to the molecular radius (*k* = 1), this corresponds to a protein of molecular weight approximately 170 kDa.

Anisotropic diffraction limits cannot be imposed in the SRF. Since data in the weakly diffracting direction will not contribute signal to the SRF, anisotropic data should be truncated isotropically at a suitably lower resolution limit when the radius of integration is constrained by the limit on *L*_max_.

### 6.3. Theoretical cumulative Patterson density

**Figure 2:**
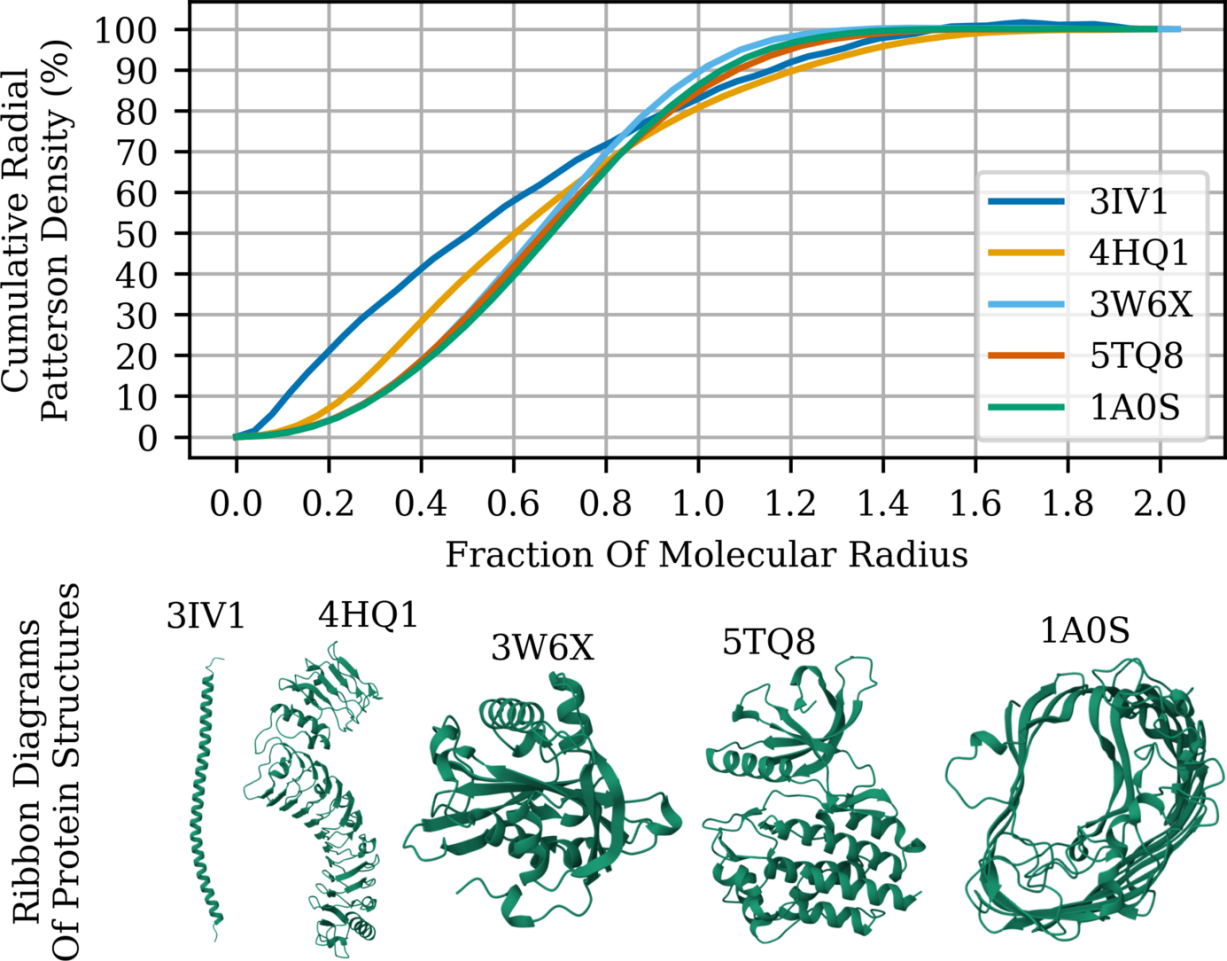
Spatial distribution of Patterson density for representative protein structures. The cumulative radial Patterson density is plotted as a function of the fractional molecular radius for five protein structures chosen to differ markedly in fold: PDB entry 3ivi is an extended α-helix extracted from a coiled coil, PDB entry 4hq1 is an extended leucine-rich repeat with two solenoids, PDB entry 3w6x is a highly globular protein, PDB entry 5tq8 is a bilobal kinase and PDB entry 1a0s is a porin. For each structure, the atomic model was centred at the origin, placed in a large cubic *P*1 unit cell and used to compute a map. The squared amplitudes of the Fourier coefficients were spherically averaged in concentric shells to yield the radial distribution of Patterson density. The cumulative sum of shell contributions, normalized to 100%, quantifies the proportion of total Patterson signal within a given radius. Ribbon diagrams of the protein structures are shown for visual reference, with PDB codes indicated. Each image was rendered using Mol* Viewer (Sehnal *et al.*, 2021 ▸).

Theoretical plots of the density of Patterson peaks as a function of a fraction/multiple of the molecular radius are shown in Fig. 2 ▸. Even with very different molecular shapes, ranging from extended structures to compact folds, the plot of the density of Patterson vectors varies most between folds at less than 0.6 times the molecular radius, converging to 70% of the maximal density regardless of fold at around 0.8 times the molecular radius and reaching 95% of the maximal density by 1.4 times the molecular radius.

### 6.4. Optimization of the radius of integration

The relationship between the radius of integration and the LESRF results was probed using the ‘P1-monomer’ and ‘P1-dimer’ data sets. The ‘P1-monomer’ data set was more suited to probing the noise-like features of the LESRF due to crystal packing and pseudo-symmetries, and the ‘P1-dimer’ data set was more suited to probing the signal-like features in the LESRF. Signal in ‘P1-monomer’ cases could only arise from internal domain duplications (if present) or extended secondary-structural elements, particularly helices, while in the ‘P1-dimer’ data cases it was the symmetry mapping one copy to the other.

**Figure 3:**
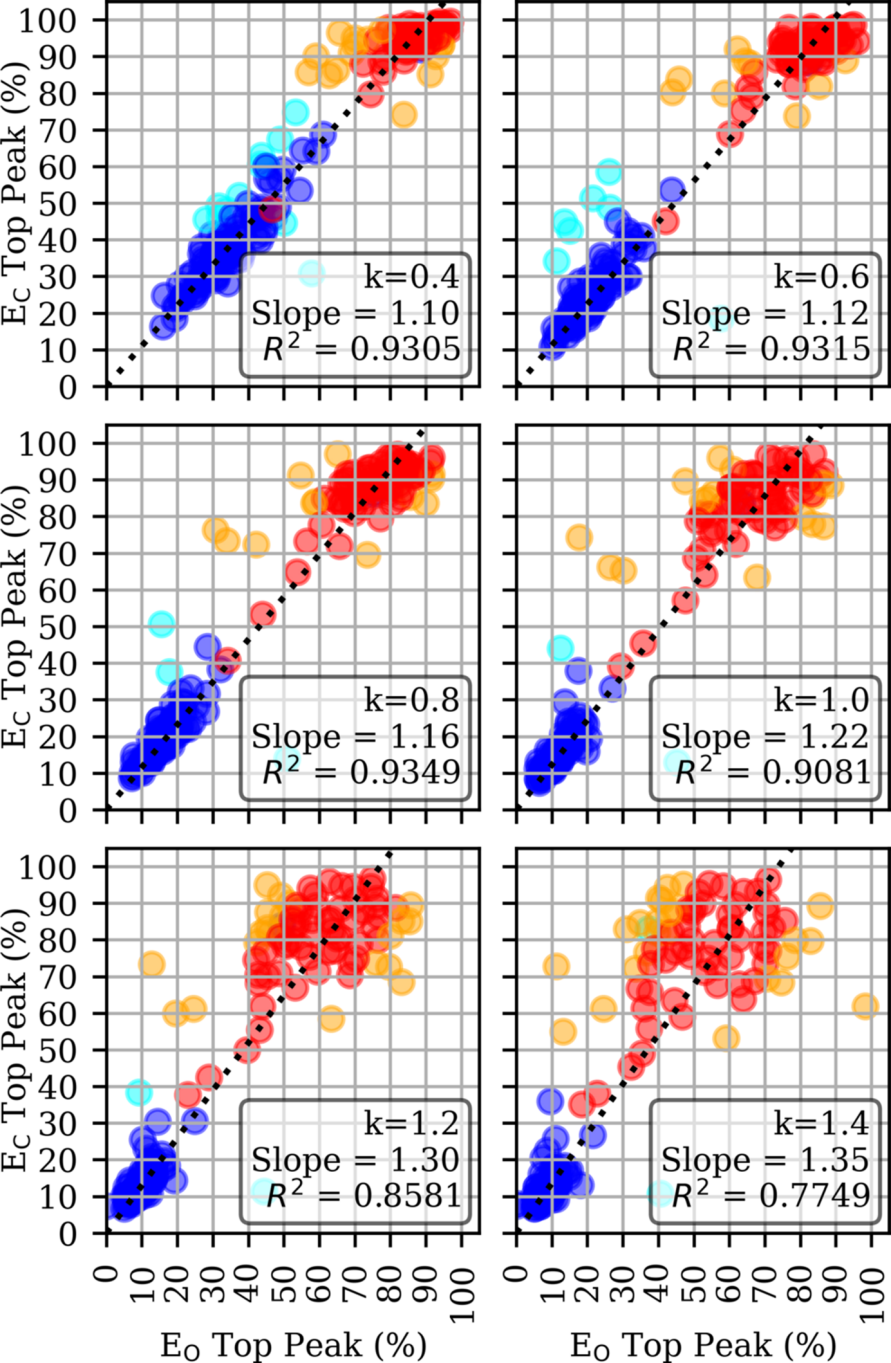
Correlation and clustering analysis of SRF peak prominence across different integration radii. Scatter plots show the relationship between the top SRF peak in calculated versus observed data sets for the ‘P1-monomer’ and ‘P1-dimer’ data sets (blue and red, respectively) across six *k* values, where *k* is the scale factor for the molecular radius *R*_M90_ (see text). Each subplot (*a*–*f*) corresponds to a different *k* value (*k* in the range 0.4–1.4 in steps of 0.2). A dashed black line indicates the least-squares regression line constrained through the origin. Outliers (points with residuals >2σ from the forced regression line) are shown in cyan (‘P1-monomer’) or orange (‘P1-dimer’).

The SRF was run using a molecular radius *R*_M90_ with the scaling factor *k* taking six values of 0.4, 0.6, 0.8, 1.0, 1.2 and 1.4. The SRF results for each test case were compared by analysing the correlation between the distributions of top SRF peak heights across cases and performing a regression analysis, as shown in Fig. 3 ▸. Three metrics, the Pearson correlation coefficient, the silhouette score and the centroid distance, were considered to optimize *k*.

**Figure 4:**
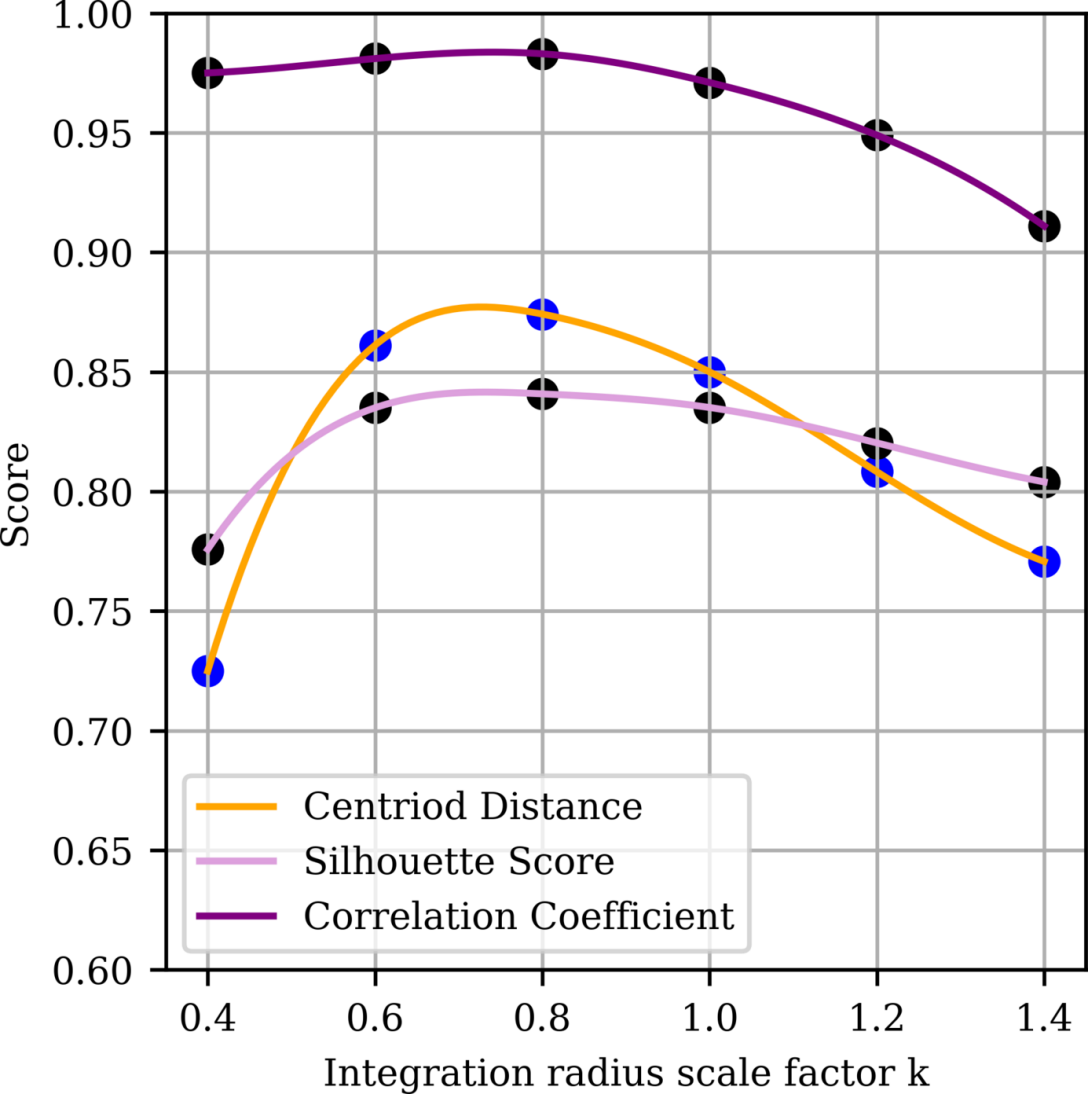
Comparison of ‘P1-monomer’ and ‘P1-dimer’ class separation metrics as a function of integration radius expressed as the scale factor *k* of the molecular radius *R*_M90_. Data are as in Fig. 3 ▸. Three scoring criteria were plotted for varying values of *k* from 0.4 to 1.4: the correlation coefficient between observed and calculated peak prominence (purple), the centroid distance between cluster centres (orange) and the silhouette score (plum), as described in the text. Curves were generated by cubic spline interpolation of six empirical values (shown as scatter points). The three metrics have no units.

Pearson correlation coefficients between the SRF highest peak height per case for the observed and calculated data per *k* showed a maximum at *k* = 0.8 (Fig. 4 ▸).

The silhouette score for a point is calculated by taking the difference of its average distance to all other points in the same cluster (cohesion) and the average distance to all points in the other cluster (separation). It takes values between 1.0 (cohesive and separated) and −1.0 (suggesting misclassification, were classification uncertain), with values of 0.0 for points on the boundary. The overall silhouette score, which is the average of all individual scores across the data set (Fig. 4 ▸), showed a maximum at *k* = 0.8.

The centroid distance is a calculated as the distance between the centroids of the clusters; this metric does not capture the spread of the clusters. The centroid distance scores per *k* showed a maximum at *k* = 0.8

The three measures agreed in indicating that the optimum was *k* = 0.8. Note that the density of Patterson peaks at *k* = 0.8 also benefits from not being particularly sensitive to molecular shape (Fig. 2 ▸). This value was used in all later analysis.

### 6.5. Applicability to other SRF implementations

The optimization determined here is relevant to other implementations of the SRF. In *CNS*, *X-PLOR* and *POLARRFN* the molecular radius is required user input. In *MOLREP*, the user can instead choose to input the molecular weight of the protein, from which a radius (method undocumented) is calculated. The *MOLREP* documentation suggests, without attribution, that an appropriate integration radius is twice the radius of gyration. Since the radius of gyration for a solid sphere of radius *r* is 0.44*r*, the suggested integration radius (for a ‘spherical’ protein) is 0.88*r*, which is close to the value of 0.8*r* found to be optimal in this study.

### 6.6. Applicability to the CRF

The signal from the CRF is degraded by inaccuracies in the model structure, while the signal from the SRF is enhanced because there is no ‘model’ inaccuracy (only the relatively minor errors in the measurement of experimental data). Conversely, the CRF signal is enhanced by the ability to remove any intermolecular vectors by calculation of the structure factors in an unphysiological crystal created such that the assemblies in the lattice are separated by over twice their molecular radii, while the SRF signal is degraded by unavoidable intermolecular vectors (since the crystal forms a connected lattice).

These highly divergent properties of the CRF versus the SRF mean that optimizations of the radius of integration *b* relevant to the SRF will not translate to optimizations of the integration radius for the CRF. Indeed, we have previously established that it is optimal to include all vectors for the CRF (Storoni *et al.*, 2004 ▸).

## 7. Asymmetric unit copy number

We aimed to improve the prior probabilities for the number of copies in the asymmetric unit by the inclusion of information from the LESRF in the case of general noncrystallographic symmetry. The data extracted from the PDB were sparse for 11 or more than 12 assemblies in the asymmetric unit. Therefore, our predictions aimed to classify the data into one of 12 classes (1, 2, 3, 4, 5, 6, 7, 8, 9, 10, 12, X), where X was for 11 or more than 12, also serving as a catch-all to accommodate uncertainty. This allowed the prediction to focus its discriminative power on the well populated classes, while still accounting for rare or ambiguous instances without compromising the overall accuracy.

### 7.1. Matthews coefficient

Prior probabilities for the number of assemblies present in the asymmetric unit have long been established from the Matthews coefficient (Matthews, 1968 ▸). Most nonviral protein structures have a protein content within 20–70% (Kantardjieff & Rupp, 2003 ▸).

The Matthews coefficient is a stringent prior when the molecular volume to asymmetric unit volume ratio is high. It is diagnostic for one copy if one copy has a protein content over 50%. If the hypothetical presence of one copy in the asymmetric unit gives less than 50% protein, in practice the protein content for one copy needs to be less than 35% to also allow two copies within the 20–70% range, and less than 23% to also allow three copies. The Matthews coefficient becomes progressively less informative about asymmetric unit copy numbers as the molecular volume to asymmetric unit volume decreases.

The solvent content of protein crystals is known to be correlated with the maximal diffraction resolution of the crystals, since tighter packing leads to higher order in the lattice. Different space groups have been investigated for correlation with different average solvent contents, with it having been observed that more frequently observed space groups have lower average solvent contents (Andersson & Hovmöller, 2000 ▸). However, a later study indicated that the important aspect of differences in solvent content between space groups was the symmetry *L* value, the number of independent parameters for describing the unit cell (Wukovitz & Yeates, 1995 ▸), and the average *V*_M_ ranges from 2.40 Å^3^ Da^−1^ for triclinic lattices to 3.44 Å^3^ Da^−1^ for cubic lattices (Chruszcz *et al.*, 2008 ▸).

In the special case of commensurate tNCS, the number of copies in the asymmetric unit can be deduced from the tNCS vectors. This is most easily performed when the vectors are expressed in fractional coordinates. For example, a single tNCS vector at (½, 0, 0) implies a multiple of two in the asymmetric unit, and the set of vectors (¼, 0, 0), (½, 0, 0) and (¾, 0, 0) imply a multiple of four in the asymmetric unit.

### 7.2. Pilot study with decision tree

Our previous work on tNCS established criteria for the recognition of tNCS from the Patterson. In summary, a native Patterson peak of 16% identified significant tNCS, where significance was defined by the need to correct the intensities for the tNCS-associated systematic intensity modulations before use in the maximum-likelihood functions for molecular replacement (Caballero *et al.*, 2021 ▸). We wished to establish a similar criterion for distinguishing between the presence and absence of general NCS using the SRF.

As a pilot study, we selected from our data set monomer and dimer cases for all space groups. Unlike the study for the optimization of the integration radius, we included all cases regardless of the root-mean-square deviation between the copies in the asymmetric unit (for cases of two in the asymmetric unit).

**Figure 5:**
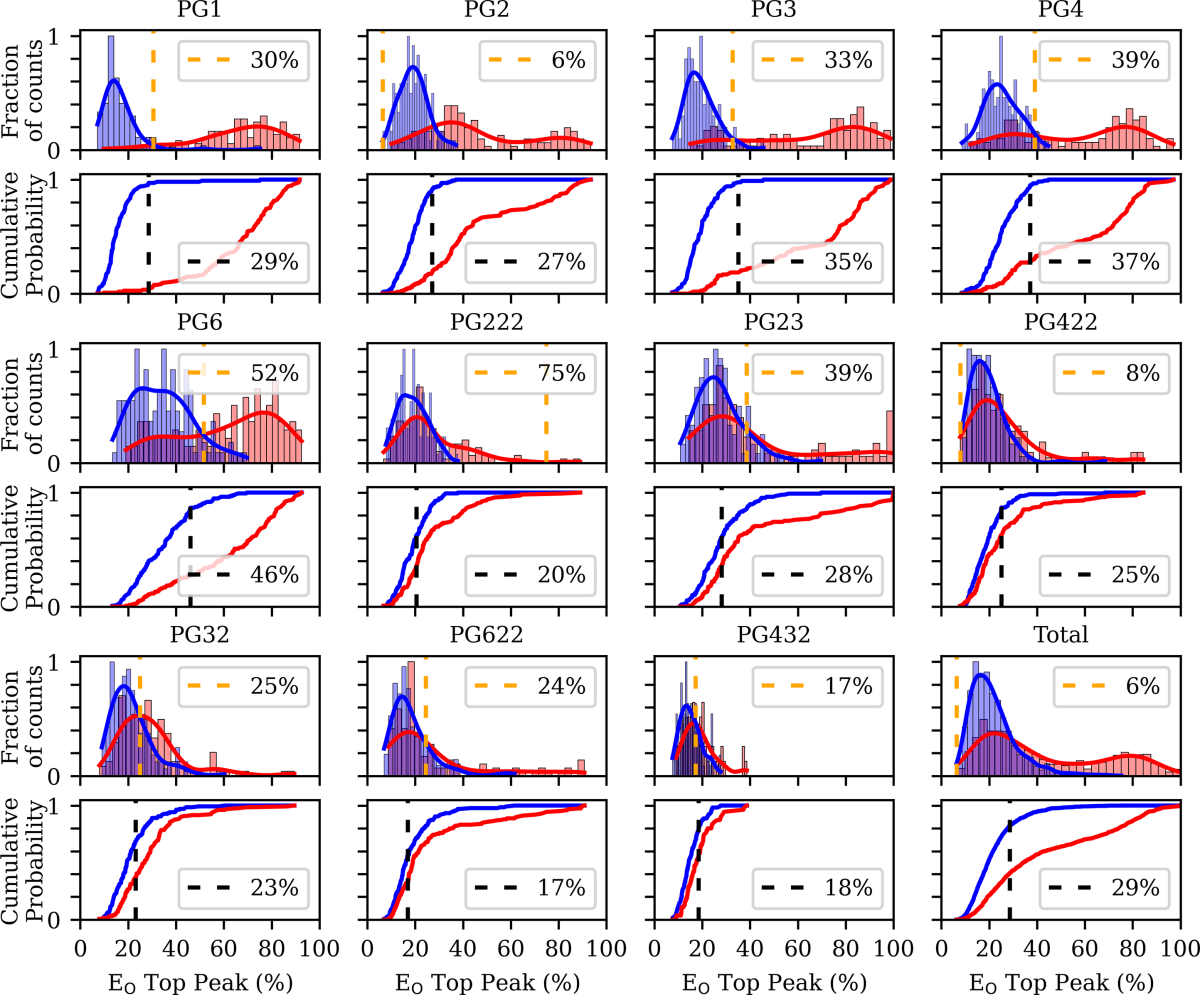
Distributions of SRF peak-height values by symmetry group and oligomeric state. Each symmetry group (PG1 to PG432, plus a combined ‘Total’) is represented by a pair of vertically stacked plots: a density-normalized histogram (top) and a cumulative distribution function (ECDF; bottom). Blue and red indicate data from the ‘P1-monomer’ and ‘P1-dimer’ data sets, respectively. In the histograms, the shaded bars show the relative frequency distributions of SRF peak-height (%) values with overlaid kernel density estimates (KDEs), each normalized to a maximum of 1. An orange dashed line marks the KDE-based threshold at which the densities of the two classes are most similar. In the ECDF plots, a black dashed line indicates the value at which the absolute difference between monomer and dimer cumulative distributions is greatest (the Kolmogorov–Smirnov statistic). Axes are shared within rows; *x*-axis labels appear only on the lower subplots and *y*-axis labels only on the leftmost panels.

Histograms of the distribution of top non-origin peak heights as a percentage of the origin (Fig. 5 ▸), were analysed to find the Kolmogorov–Smirnov (KS) threshold, the point on the cumulative distribution curves where the separation between classes is maximized, which is the decision boundary where the true-positive rate and the false-positive rate differ the most.

The ability to distinguish one from two in the asymmetric unit (two classes for categorization) became progressively more difficult as the number of crystallographic space-group operations increased. For more than six symmetry operations, the SRF top peak-height distributions for the two classes overlapped extensively. No peak-height cutoff gave good discrimination between the two classes.

Attempts to add other criteria to the classification, from amongst the many in the database associated with the data set, via a decision tree failed to find any combination of criteria that improved the ability to distinguish classes (data not shown).

The failure to find a SRF peak height diagnostic for the presence of general noncrystallographic symmetry even in this simple pilot study indicated that this decision-tree approach would not straightforwardly yield helpful insights when extended to higher order noncrystallographic symmetry.

### 7.3. Circular, dihedral, octahedral and icosahedral symmetry

**Figure 6:**
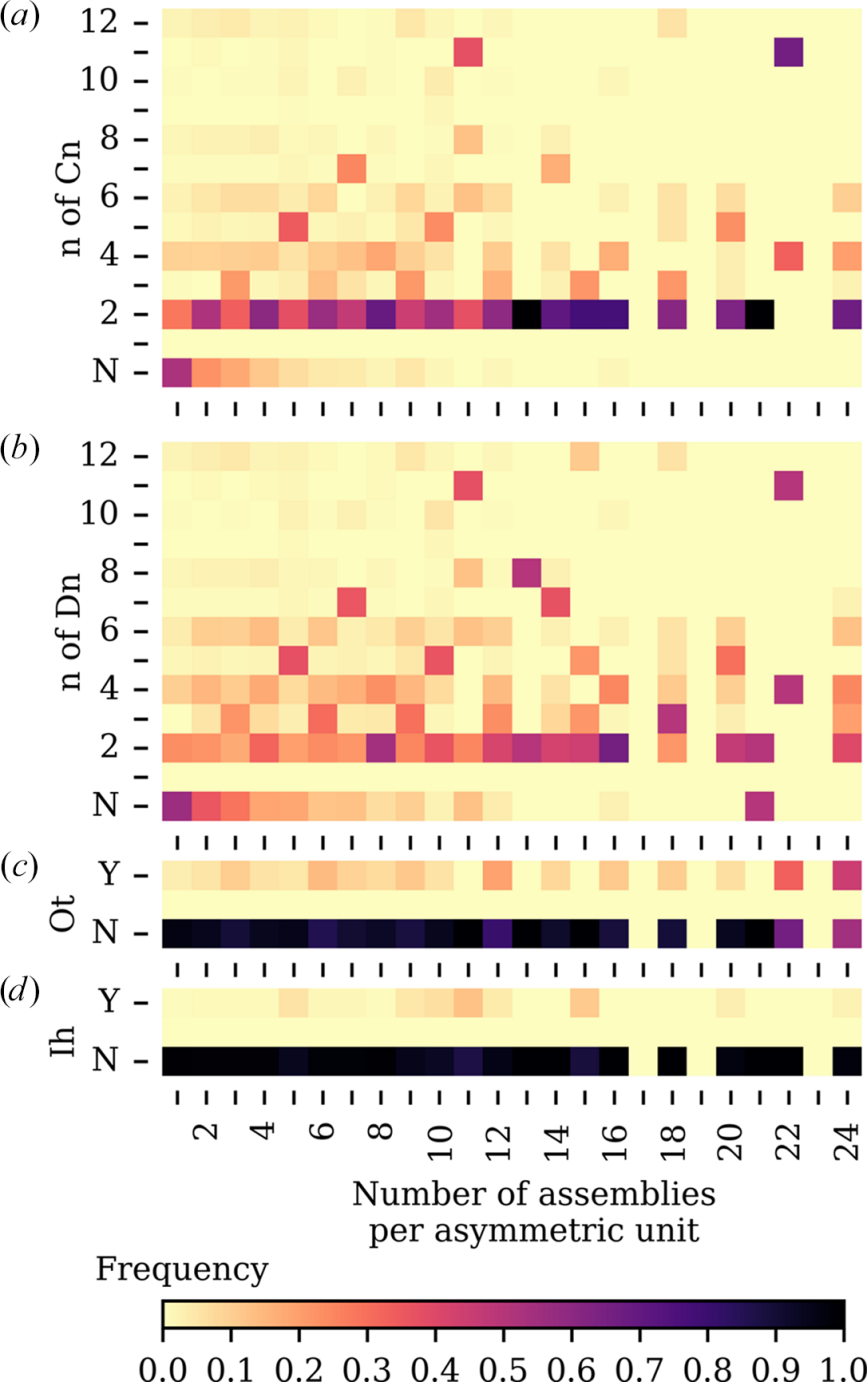
Normalized frequency heatmaps of SRF symmetry indicators by number of assemblies in the asymmetric unit. Each panel shows a 2D histogram of symmetry-related metrics (*y* axis) as a function of the number of assemblies per asymmetric unit (*x* axis). (*a*) *n* of the point group *C_n_* (*y* axis) for the most prominent peak over 20% of the origin peak, with ‘N’ indicating no peak over 20% of the origin. (*b*) *n* of the point group *D_n_* (*y* axis) for the most prominent peak over 20% of the origin peak, with ‘N’ indicating no peak over 20% of the origin. (*c*) Presence of an octahedral symmetry with all peaks for the set of symmetry operators over 20% of the origin. (*d*) Presence of an icosahedral symmetry with all peaks for the set of symmetry operators over 20% of the origin. Colour intensity indicates the relative frequency (0–1) within a given column.

The relationship between the number of copies of the assembly in the asymmetric unit components and the presence of circular, dihedral, octahedral and icosahedral symmetry in the SRF in our data set is shown in the series of heatmaps in Fig. 6 ▸. The data are presented in a stacked bar chart in Supplementary Fig. S6.

The most common SRF symmetry is *C*_2_, regardless of the number of copies in the asymmetric unit. The presence of *C*_2_ symmetry therefore contains little information about the contents of the asymmetric unit. The presence of odd *n* integer *C*_*n*_ symmetry as the top peak in the SRF, for example *C*_5_, *C*_7_ or *C*_11_, is much more informative about the number of copies in the asymmetric unit.

Where *C*_2_ symmetry is present, it can also be a subgroup of higher order oligomers, for example tetramers that are dimers of dimers with *D*_2_ symmetry. Where *C*_*n*_ symmetries are present as a subgroup of *D*_*n*_ symmetry, they may be considered significant even if the *C*_*n*_ peak is not the highest in the SRF: commonly it is the associated perpendicular *C*_2_ symmetry that has the highest SRF peak. The presence of odd-integer *D*_*n*_ symmetries, for example *D*_5_, *D*_7_ or *D*_11_, can be diagnostic for the number of copies in the asymmetric unit even if the associated *C*_*n*_ is not the highest SRF peak.

Octahedral symmetry (*O*_t_), with 24 positions related by rotational symmetry, is rarely found in the SRF in our data set, except in the case of 24 copies of the assembly in the asymmetric unit.

Icosahedral symmetry (*I*_h_), with 60 positions related by rotation symmetry, was found at very low incidence in the SRF in our data set, as our data set did not include (icosahedral) virus structures.

### 7.4. Methods for machine learning

The differences in *C*_*n*_, *D*_*n*_, *O*_t_ and *I*_h_ symmetry detected in the SRF between different asymmetric unit copy numbers indicates that there is information in the SRF that could be interpreted by machine learning (Fig. 6 ▸, Supplementary Fig. S6).

The data were organized into a three-tiered directory structure to facilitate reproducible loading and splitting. At the top level, samples were divided into ‘train’, ‘validate’ and ‘test’ sets with an approximate 80%/10%/10% split, stratified to balance the SGPG distribution as far as possible. Classes for only one or two copies in the asymmetric unit were further restricted to avoid vast overrepresentation (Table 1 ▸). Within each split, second-level subdirectories were named for the integer number of biological assemblies per asymmetric unit and third-level subdirectories were named for individual PDB identifiers. Each such PDB folder held exactly 13 112 × 112-pixel greyscale images in portable network graphics (PNG) format, representing successive slices of the self-rotation function as described above, with the first image being the ‘Matthews-data’ image and images 2–13 being the 12 SRF images. Classes with fewer than 100 samples were pooled into a single ‘X’ category, serving both to stabilize training and to capture out-of-distribution cases.

Images were streamed on the fly using a custom generator that loads each of 13 greyscale PNG files per sample, stacks them along the depth axis to produce a 3D tensor of shape (112, 112, 13) and pairs each tensor with a one-hot-encoded class label based on directory structure. This generator was wrapped in a TensorFlow Dataset pipeline configured for high-throughput execution on a 120-core CPU node under the CentralStorageStrategy. The pipeline applies per-image standardization, shuffling for training and validation sets, batching (32 samples per batch) and asynchronous prefetching to maximize efficiency.

The classification model was implemented in Keras using the Sequential API as a pruned 3D convolutional neural network. It comprises three Conv3D layers with increasing filter depths (32, 64, 128), all using 3 × 3 × 3 kernels, ReLU activations and ‘same’ padding. Each Conv3D layer was followed by MaxPooling3D that pools over the two spatial dimensions but preserves depth. The resulting 3D feature volume was flattened and passed through a 256-unit dense layer with ReLU activation and a dropout rate of 0.5, before being projected by a final dense layer with ‘softmax’ activation onto the 12 output classes.

To reduce model size and improve efficiency, layer-wise magnitude-based pruning was applied to the convolutional layers using the *TensorFlow Model Optimization Toolkit*. A polynomial pruning schedule progressively increased sparsity from 0% to 50% over the course of training. The network was trained for up to 25 epochs using the Adam optimizer with an initial learning rate of 1 × 10^−4^, subject to a custom learning-rate scheduler that reduced the rate at epochs 5, 10, 15 and 20. Early stopping with a patience of five epochs monitored validation loss and restored the best weights. Model selection was based on validation accuracy rather than loss. After training, pruning wrappers were stripped to yield a deployable, compact model, which was saved in HDF5 format (320 Mb).

During the evaluation phase, the performance of the model was measured using test data that were not seen during training. Performance was evaluated via overall accuracy, a classification report of precision, recall, F1 score and support, and a confusion matrix.

### 7.5. Control study

As a control, the Matthews coefficient was encoded as a graphic, as above, but with no other information from the SRF (Supplementary Fig. S4). The control served as a baseline for analysing the architecture of the training on the predictions. The convolutional neural network architecture was adapted from the primary study, with the key difference that only a single greyscale image was loaded per sample, and the three convolutional layers were implemented as 2D convolutions (Conv2D) rather than 3D (Conv3D).

### 7.6. MLE-Matthews analysis of asymmetric unit contents

**Figure 7:**
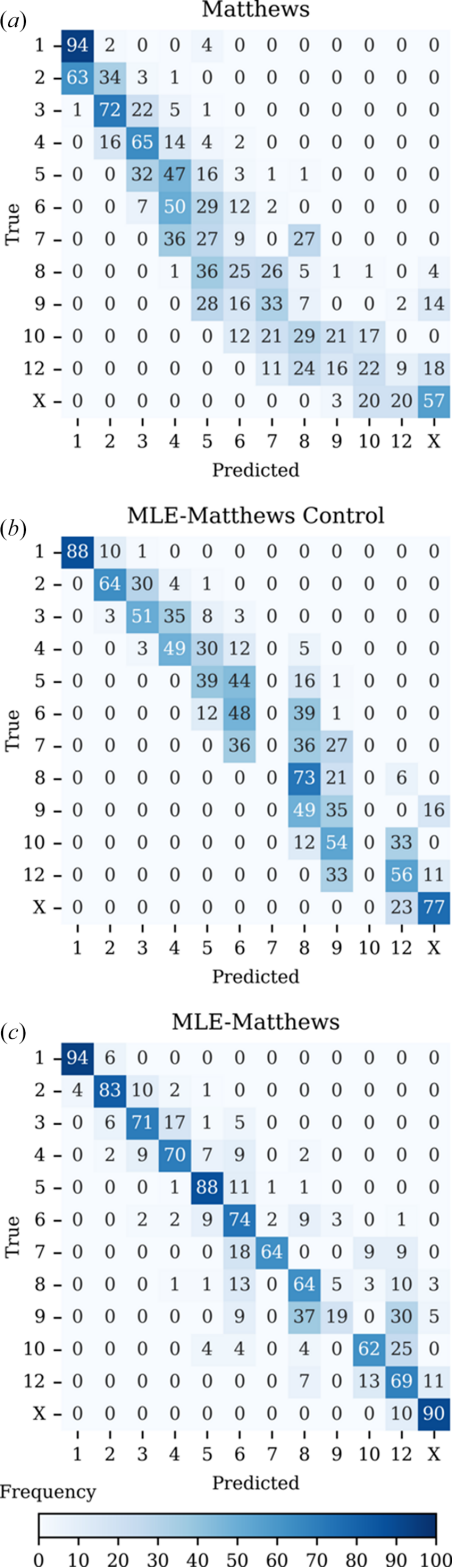
Normalized confusion matrices comparing the classification performance of the MLE-Matthews model against the Matthews model on test data. The heat maps show per-class prediction distributions for 12 asymmetric unit categories (1–10, 12 and X). Rows represent the true classes and columns the predicted classes, with values normalized by row to highlight recall performance. Colour intensity corresponds to the percentage of predictions within each true class, with darker shades indicating higher proportions. (*a*) The Matthews model displays widespread off-diagonal elements, indicating substantial misclassification, particularly as the number of copies increases. (*b*) The MLE-Matthews Control model also displays widespread off-diagonal elements, indicating substantial misclassification. (*c*) The MLE-Matthews model has strong diagonal dominance, reflecting the accurate and specific classification of structural classes.

The results of the machine learning are compared with the Matthews coefficient by asymmetric unit copy number in Table 2 ▸ and by space-group point group in Table 3 ▸. Confusion matrices of the training are shown in Fig. 7 ▸. The overall accuracy for the Matthews model was 0.44, that for the MLE-Matthews Control model was 0.67 and that for the MLE-Matthews model was 0.81.

The MLE-Matthews Control model improves on the Matthews model for two reasons. While the Matthews model predicts unconstrained integer values representing the number of assemblies per asymmetric unit, the MLE-Matthews Control model is constrained to select only from the predefined set of allowed classes. This constraint acts as a form of regularization, inherently reducing the chance of erroneous predictions. Additionally, because the predefined classes reflect the empirical distribution of training data, the constraint introduces an implicit prior: more frequent classes are favoured, biasing the classifier toward commonly observed copy numbers. Consequently, the MLE-Matthews Control model achieves higher F1 scores than the Matthews model, despite no new information being added. Performance differences should therefore be interpreted as structural advantages. There is no structural difference between the MLE-Matthews Control and MLE-Matthews models.

The MLE-Matthews model consistently outperformed the MLE-Matthews Control model. Accuracy was lowest for the higher symmetry space groups, as expected from the pilot study for monomers and dimers.

The inference stage applied a hard limit on the classification dependent on the maximum possible for the asymmetric unit volume. If the predicted class was outside the limit, the highest probability class within limits was used. However, none of the test cases triggered this fallback, which may reflect the effectiveness of the training.

## 8. Discussion

The SRF has sat rather uncomfortably amongst the pantheon of crystallographic data-analysis tools. Constantly recommended as a ‘first-look’ method, it has never been systematically incorporated into structure-solution pipelines, and there are no well defined rules as to what the crystallographer is to do with what it reveals. The SRF has seemed best interpreted in the rear-view mirror, with the relationship of the SRF peaks to arrangements within the asymmetric unit and crystallographic symmetry operations only discernible *post hoc*.

A nice example is reported in the *CCP4 Newsletter* article *A Self-Rotation Puzzle* (Cao & Isaacs, 2007 ▸), which explores the crystal structure of bovine mitochondrial peroxiredoxin III (PrxIII), an antioxidant enzyme involved in regulating intracellular hydrogen peroxide levels. Other species of PrxIII had been found to form pentameric rings of dimers (decamers; Alphey *et al.*, 2000 ▸). The Matthews coefficient for their novel bovine PrxIII crystal form in space group *C*2 suggested between five and 12 dimers in the asymmetric unit. The SRF showed clear patterns of twofolds, but did not indicate the expected fivefold. Rather, there were two sixfold axes, one twofold stronger than the others, and a rotation of 55°. The authors were unable to decipher the SRF *a priori*. Upon phasing by molecular replacement, the crystal structure revealed two half-rings in the asymmetric unit, giving interlocking dodecameric rings in the unit cell, a protein catenane where two closed rings are linked without covalent bonds, which is highly unusual in protein structures. The SRF could then be interpreted as two sixfolds perpendicular to the planes of the rings, a family of twofolds perpendicular to the ring axes and a 55° rotation corresponding to the inclination of the rings (Fig. 1 ▸; Supplementary Figs. S1 and S2). This remains a difficult case, with the MLE-Matthews model giving the highest probability (0.89) in the X (other/unsure) category.

Attempts have been made to incorporate the symmetries detected in the SRF to enhance the CRF, although the method has not gained traction. The locked rotation function (Tong & Rossmann, 1990 ▸), implemented in the programs *GLRF* and *MOLREP*, aims to take advantage of the symmetry identified in the SRF to average the CRF to reduce noise. A locked rotation function has not been implemented in our software *Phaser* or *Phasertng*, not least because any advantage bestowed by the averaging is diminished by errors in the SRF, and our alternative signal-enhancing method of rescoring CRF-identified rotations with a full maximum-likelihood rotation function (Read, 2001 ▸) is extremely effective and accounts for errors.

The dominance of *C*_2_ symmetry in the SRF across all space groups and asymmetric unit contents is not surprising. Proteins commonly evolve dimerization because it is a simple way to evolve stable and versatile function, for example to enable a single protein to act as a switch by toggling its monomeric/dimeric state, or to transmit conformational change for cooperative binding. A weak tendency to self-associate might only require a few mutations to form a stable *C*_2_ dimer, since the dyad symmetry means that advantageous mutations on one half of the interface are also advantageous at the symmetrical site. The total free-energy gain includes enthalpy terms from the formation of electrostatic and hydrogen bonds in the interfaces, and the entropic effect of the exclusion of water, but is reduced by the dimerization itself, which imposes an entropic cost as the two freely diffusing monomers become a dimer. However, if the buried surface area is large enough, typically for a total buried surface of more than 2000 Å^2^, this energetic trade-off tips in favour of dimerization (Chothia & Janin, 1975 ▸).

Although the *C*_2_ dimers present in a crystal lattice may or may not form under physiological conditions, the same symmetrization of energetic and entropic properties that underlie the formation of biological C_2_ dimers also underlie the formation of nonphysiological *C*2 dimers facilitated by the high protein concentration environment of the crystallization drop.

Higher order *C*_*n*_ oligomers require more complex evolutionary pathways to become stable, since each binding surface is not bilaterally symmetric. While the entropy gain by excluding water from each interface increases with the number of subunits in an oligomer, the entropy cost of association also rises with each additional subunit. Higher order oligomers thus impose further entropy penalties without necessarily providing proportionally greater stabilization for the same buried surface area. *C*_2_ dimers hit the sweet spot, offering a large energetic gain at a minimal entropic cost. Higher order *C*_*n*_ oligomers are also difficult to stabilize in a partly associated state and so carry a greater risk of aggregation and protein insolubility, handicapping any evolutionary advantage. Thus, if *C*_*n*_ symmetries with *n* > 2 are seen in the SRF, they are more likely to correspond to successfully evolved, biological oligomer states.

The propensity to form dimers also manifests in that higher order *C*_*n*_ symmetry commonly present as a subgroup of *D*_*n*_ symmetry (Fig. 6 ▸). This appears to particularly be the case where *n* takes the odd values 5, 7 and 11. These symmetries are incorporated in the machine learning, but inspection of the SRF plots by the crystallographer will remain an important step in identifying these more unusual symmetries.

Lower symmetry space groups (such as *P*1) permit maximal molecular freedom in packing and thus often give tight packing. As symmetries are added, so too are constraints on how the molecules in the lattice must form layered or helical associations. Protein packing around twofold symmetry axes generally involves ‘bump-to-bump’ interactions, while screw axes allow ‘bump-to-hollow’ interactions (Filippini & Gavezzotti, 1992 ▸): ‘bump-to-bump’ interactions tend to enlarge voids (higher solvent). Empirically, high packing density correlates with higher diffraction resolution. Therefore, differences in the average Matthews coefficients for different Bravais lattices (Chruszcz *et al.*, 2008 ▸) are likely to already be (indirectly) considered for the purposes of asymmetric unit estimation through the resolution dependence of the Matthews probability estimates (Kantardjieff & Rupp, 2003 ▸; Weichenberger & Rupp, 2014 ▸). Since the space group is a hypothesis in ‘first-look’ analysis, particularly regarding the presence or absence of screw axes along any given axis, we have chosen not to incorporate the annotation of the screw symmetries of the refined structure’s space group into our training.

Incorporating information about oligomeric state into structure-prediction algorithms can enhance the accuracy of the resulting models, particularly at the interfaces between subunits, which are often highly conserved (Jumper *et al.*, 2021 ▸). Predicting a protein in the monomeric state when it naturally forms an oligomer may lead to an incorrect fold, because the algorithm may want to place hydrophobic amino acids that should be solvent-accessible in the hydrophobic interior. Tools such as *AlphaFold-Multimer* (Evans *et al.*, 2022 ▸; Bryant & Noé, 2024 ▸) can leverage known oligomeric states to guide the prediction of both intrasubunit and intersubunit arrangements, providing more biologically relevant structural models. However, there is currently no way of specifying the point group of the oligomer in AI-generated structure-prediction resources, so that although it cannot act as a constraint on prediction, it can be used to select more probable candidates from multiple predictions with different seeds.

The machine learning-enhanced (MLE-Matthews) method developed here is the latest in a series of enhancements to the original Matthews coefficient (Matthews, 1968 ▸; Kantardjieff & Rupp, 2003 ▸; Weichenberger & Rupp, 2014 ▸). These studies recalibrated the coefficient over thousands of data sets versus the original few hundred, added separate estimates for protein–nucleic acid complexes and, importantly, included the maximum resolution of the data in the estimate. These enhancements are implicitly included in the MLE-Matthews coefficient through being encoded in the non-SRF ‘Matthews-data’ image.

The new approach integrates features extracted from diffraction data via the LESRF. Our exploration of the peak heights of the LESRF indicated that although the LESRF is not always interpretable *a priori*, on occasions it can be, and this information can be leveraged to increase the likelihood of correctly predicting certain asymmetric unit compositions. The increase in overall accuracy from 0.44 for the Matthew estimation, to 0.67 for the MLE-Matthews Control method and to 0.81 for the MLE-Matthews method is gained mostly from improvements in predictions for higher asymmetric unit copy numbers and lower symmetry space groups, including space groups *P*2_1_2_1_2_1_, *P*2_1_ and *C*2, space groups that together account for 50% of protein crystal structures (Gaur, 2021 ▸): these are also the most useful set of circumstances. As such, this tool offers crystallographers an improved starting point for molecular replacement or experimental phasing, potentially reducing trial-and-error cycles and expediting structure solution.

The MLE-Matthews method is incorporated in *Xtricorder* and the wider *Phasertng* software package. The results should be used in conjunction with inspection of the SRF plots and tNCS analysis to corroborate how the MLE-Matthews method is drawing out likely asymmetric unit compositions. Only inspection of the SRF plots will provide specific information about the direction of rotation axes and their relationship to each other. Any other complementary lines of evidence such as biophysical studies or known oligomerization state of homologues should always be considered for their support of, or challenge to, the computational predictions, especially if being relied on for AI-generated structure prediction and/or crystallographic phasing.

## 9. Related literature

The following reference is cited in the supporting information for this article: Cao *et al.* (2005 ▸).

## Supplementary Material

Supplementary Figures. DOI: 10.1107/S2059798325009647/has5001sup1.pdf

## Figures and Tables

**Table 1:** Number of test cases for classification training, validation and testing by assemblies per asymmetric unit This table presents the distribution of test cases across the training, validation and testing sets for each class. The number of available data sets in the PDB restricts the number of samples for some classes. The training, validation and testing sets are used to train, tune hyperparameters and evaluate the performance of the TensorFlow CNN model.

Assemblies per asymmetric unit	No. in ‘train’ set	No. in ‘validate’ set	No. in ‘test’ set
1	596	60	976
2	616	60	957
3	664	68	325
4	644	62	603
5	778	142	152
6	740	80	117
7	123	8	11
8	564	67	77
9	149	26	43
10	196	21	24
12	378	56	45
X	244	27	30
Total	5692	677	3360

**Table 2:** F1 scores for predicted assemblies per asymmetric unit The table compares F1 scores across three models: the original Matthews model, the MLE-Matthews Control model (trained without SRF information) and the MLE-Matthews model (trained with SRF information). Each row corresponds to a specific number of assemblies per asymmetric unit (1–10, 12, other), with the final column indicating class support (the number of test samples for each class). The MLE-Matthews model consistently outperforms, particularly for underrepresented or previously poorly classified classes such as 5, 6, 7 and 10. The ‘other’ class refers to test samples with 11 or more than 12 in the asymmetric unit.

	F1 score			
Assemblies per asymmetric unit	Matthews	MLE-Matthews Control	MLE-Matthews	Support
1	0.74	0.93	0.95	976
2	0.40	0.77	0.86	957
3	0.16	0.47	0.65	325
4	0.20	0.56	0.76	603
5	0.16	0.27	0.76	152
6	0.16	0.40	0.55	117
7	0.00	0.00	0.67	11
8	0.07	0.48	0.58	77
9	0.00	0.00	0.27	43
10	0.18	0.00	0.61	24
12	0.14	0.39	0.57	45
Other	0.52	0.59	0.82	30
Macro average	0.23	0.41	0.81	3360
Weighted average	0.40	0..68	0.67	3360
Accuracy	0.44	0.67	0.81	3360

**Table 3:** Accuracy in predicted assemblies per space group The table compares accuracy across two models: the MLE-Matthews Control model (trained without SRF information) and the MLE-Matthews model (trained with SRF information). Each row corresponds to a specific space-group point group, with the final column indicating class support (the number of test samples for each class).

	Accuracy		
Space-group point group	MLE-Matthews Control	MLE-Matthews	No.
1	0.73	0.87	274
2	0.74	0.86	350
3	0.71	0.86	247
4	0.67	0.83	218
6	0.62	0.77	252
222	0.76	0.84	1013
23	0.61	0.78	153
422	0.59	0.73	286
32	0.50	0.72	266
622	0.49	0.73	197
432	0.39	0.75	104
Total	0.67	0.81	766

## Data Availability

*Xtricorder* and *Nomad* will be made available through the *Phenix* and *CCP*4 software packages.
