# Supplementary material for: Xtricorder: a likelihood-enhanced self-rotation function and application to a machine learning-enhanced Matthews prediction of asymmetric unit copy number
Source: Acta Crystallogr D Struct Biol. 2025 Nov 26;81(Pt 12):678–92. doi: 10.1107/S2059798325009647 (PMC12809497; doi:10.1107/S2059798325009647)
Supplement: Supplementary file 1 [file d-81-00678-sup1.pdf]

STRUCTURAL BIOLOGY

Volume 81 (2025)

Supporting information for article:

Xtricorder: a likelihood-enhanced self-rotation function and application to a machine-learning enhanced Matthews prediction of asymmetric unit copy number

Airlie J. McCoy and Randy J. Read

Figure S1 Structure of bovine mitochondrial peroxiredoxin III (PDB ID: 1ZYE (Cao et al., 2005)), a protein catenane comprising two interlocked dodecameric toroids. The assembly crystallizes in space group C2 and the images show half of the unit cell. The two rings are depicted in orange and teal to emphasize their topological interlinking. (a) View along the crystallographic x-axis, highlighting the central cavities of the toroids. (b) View along the y-axis, showing the 55° inclination of the rings. Molecular surfaces were rendered using *Mol* Viewer* (Sehnal et al., 2021).

a)

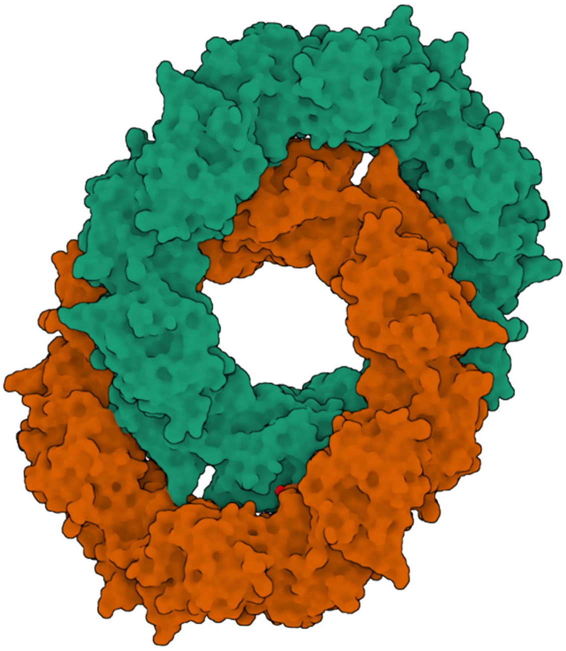

b)

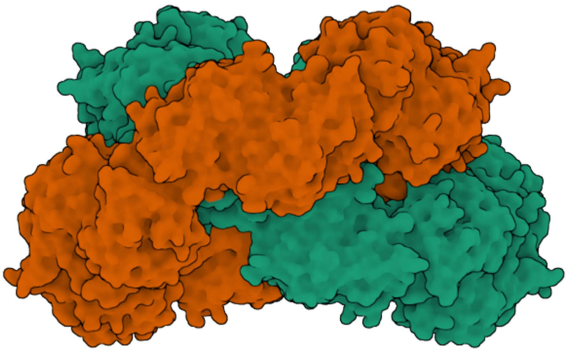

Figure S2 Composite-section diagrams for 1ZYE showing Mercator and stereographic projections of the SRF peak heights.

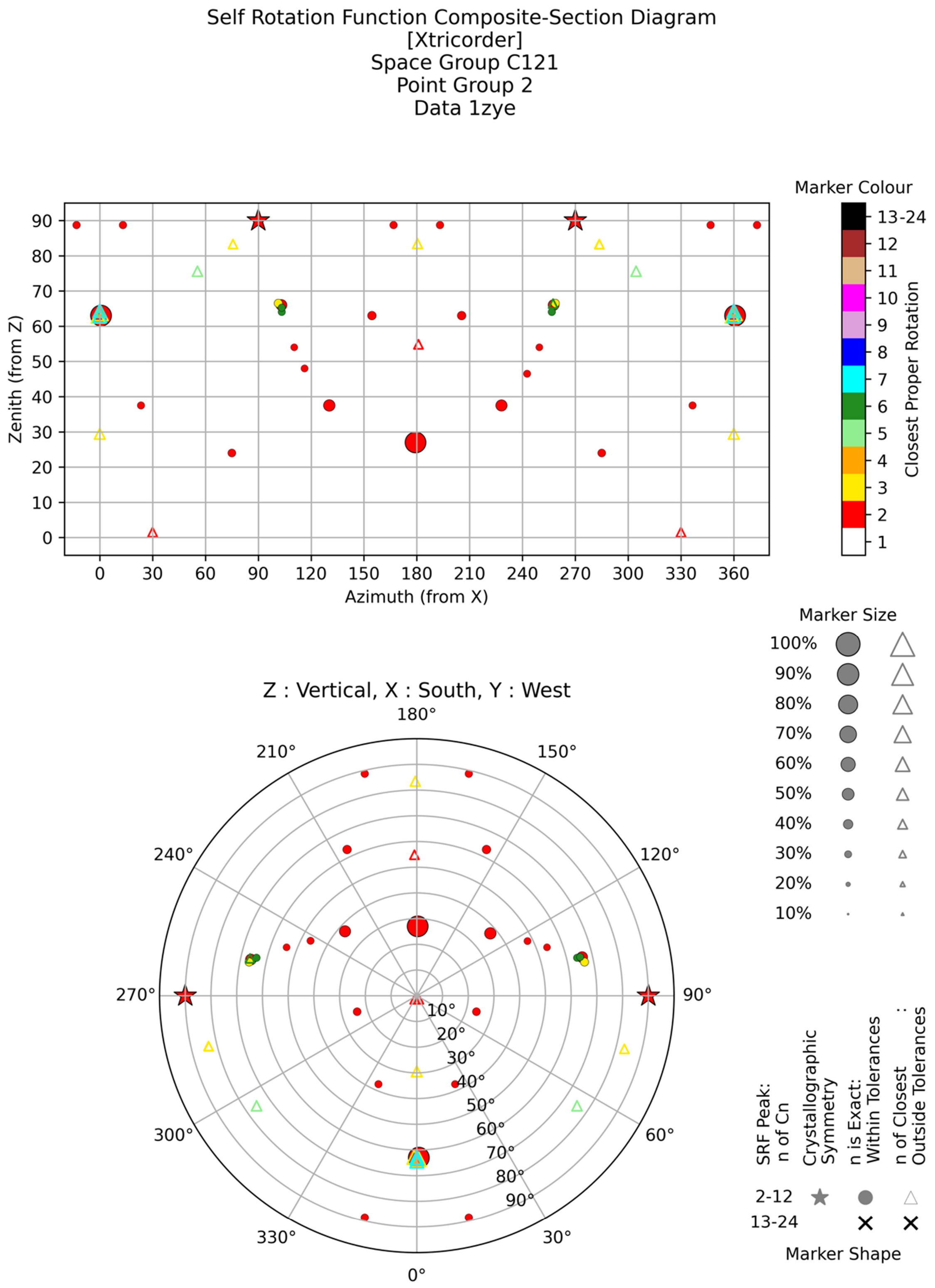

Figure S3 Self-Rotation Function (SRF) Contour Plots. Each panel (a–h) shows a 2D contour representation of a SRF section taken at a specific κ-section, selected to correspond to characteristic n-fold rotational symmetries, with $\kappa \approx 360°/n$ for $n = 2$ to 9 [180°, 120°, 90°, 72°, 60°, 51°, 45°, 40°]. The scatter plot is overlaid.

a)

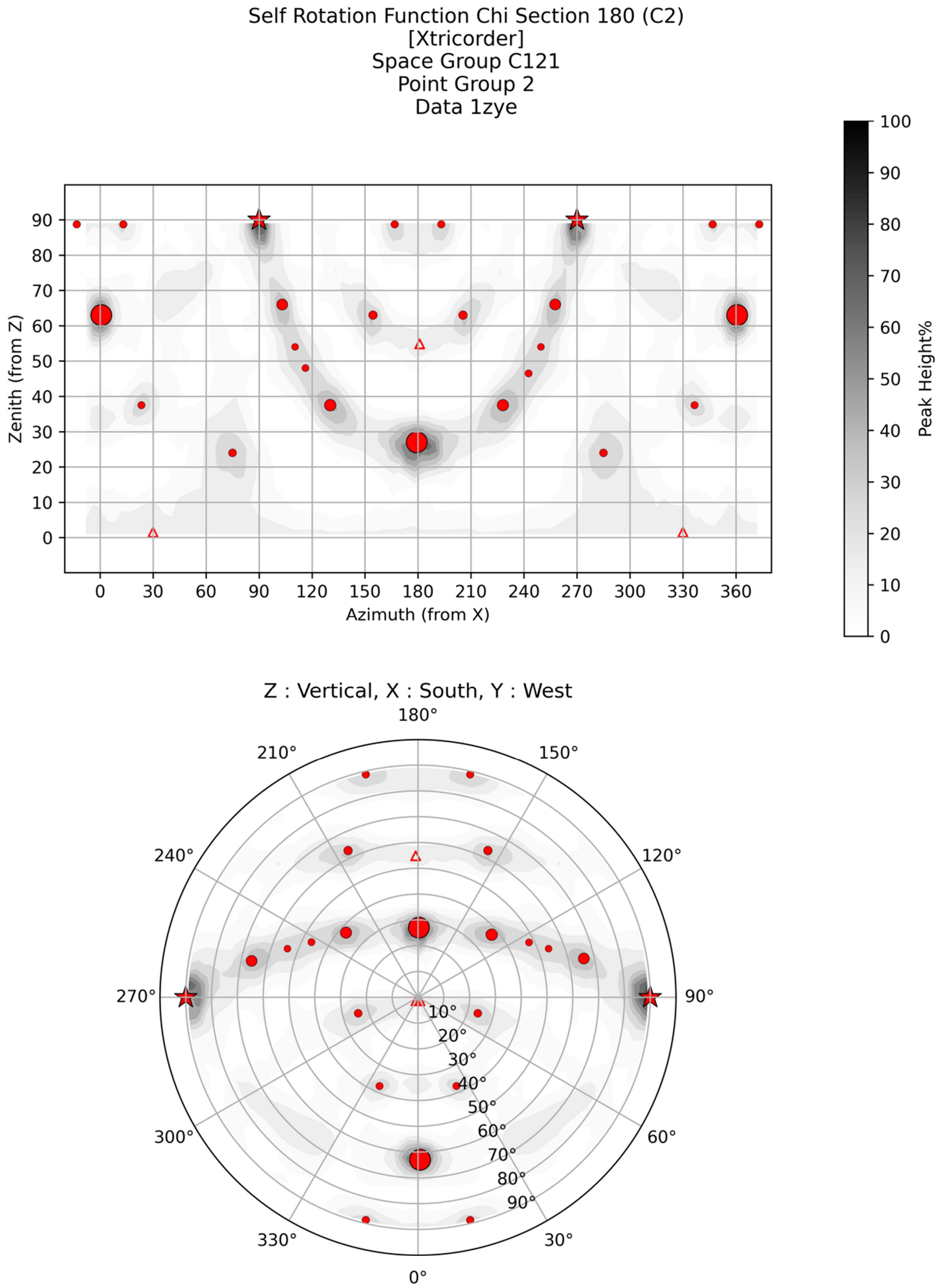
Self Rotation Function Chi Section 180 (C2)
[Xtricorder]
Space Group C121
Point Group 2
Data 1zye
Zenith (from Z)
Azimuth (from X)
Peak Height%
Z : Vertical, X : South, Y : West

b)

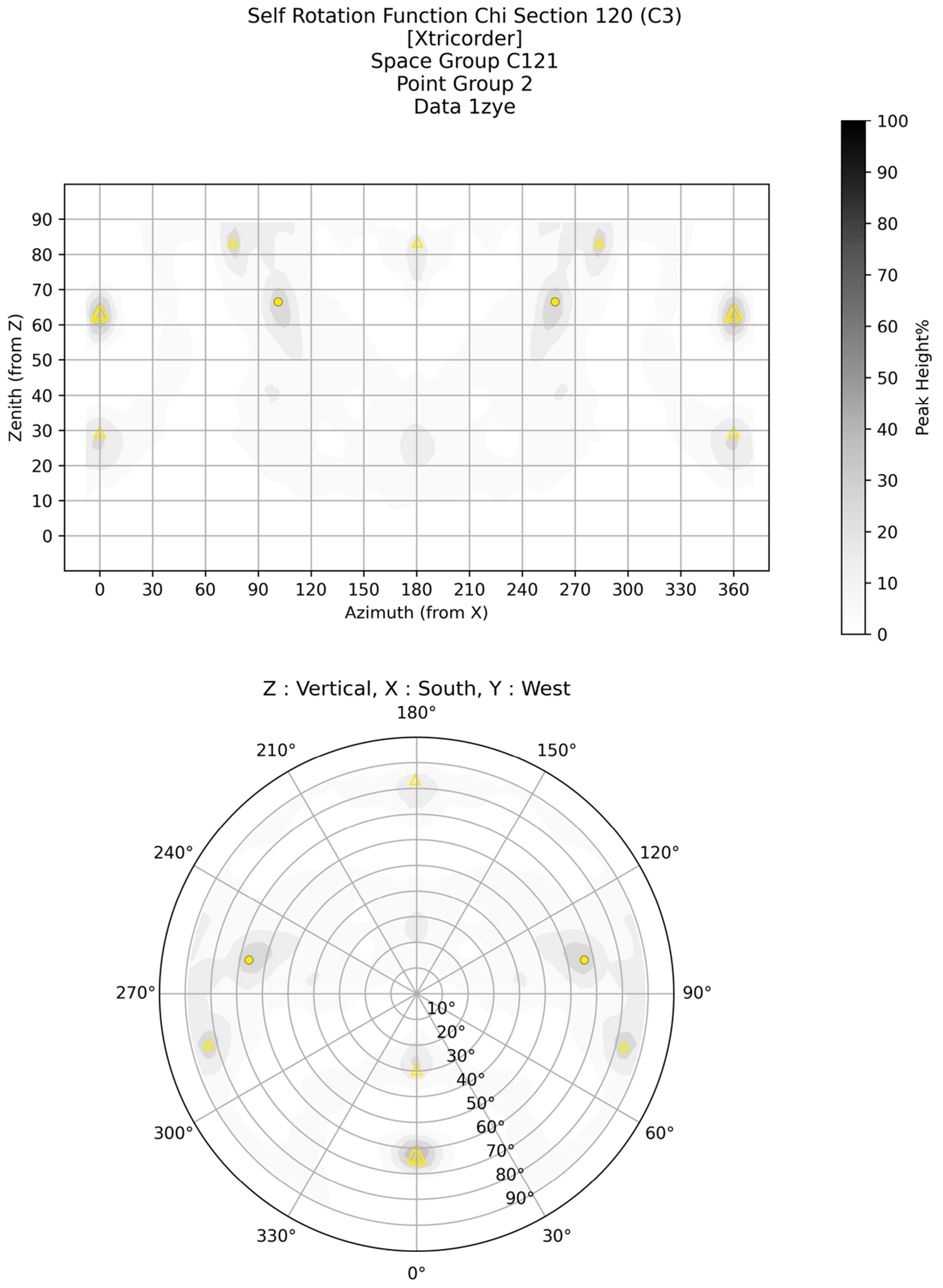
Self Rotation Function Chi Section 120 (C3)
[Xtricorder]
Space Group C121
Point Group 2
Data 1zye
Zenith (from Z)
Azimuth (from X)
Peak Height%
Z : Vertical, X : South, Y : West

c)

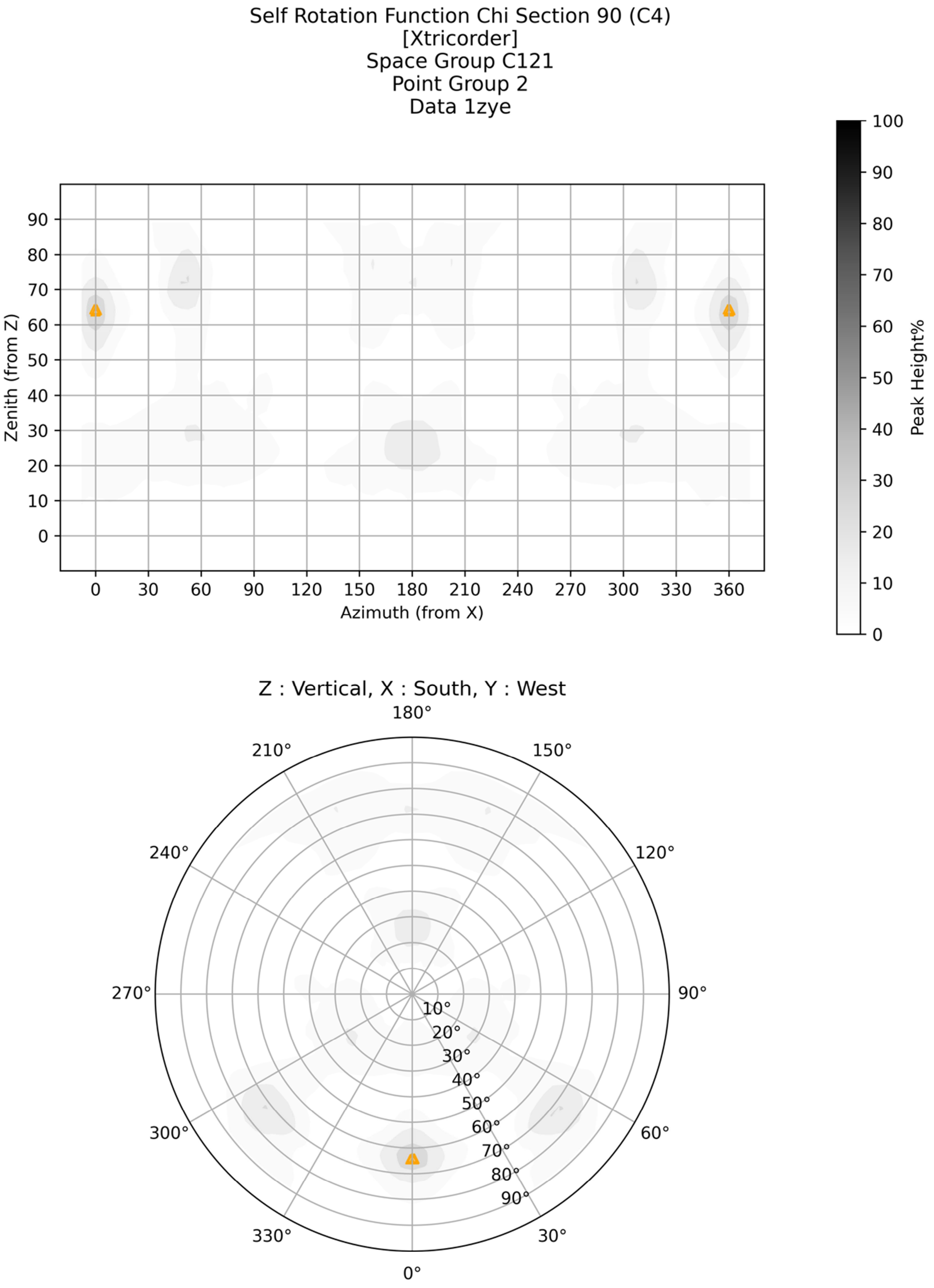
Self Rotation Function Chi Section 90 (C4)
[Xtricorder]
Space Group C121
Point Group 2
Data 1zye
Zenith (from Z)
Azimuth (from X)
Peak Height%
Z : Vertical, X : South, Y : West

d)

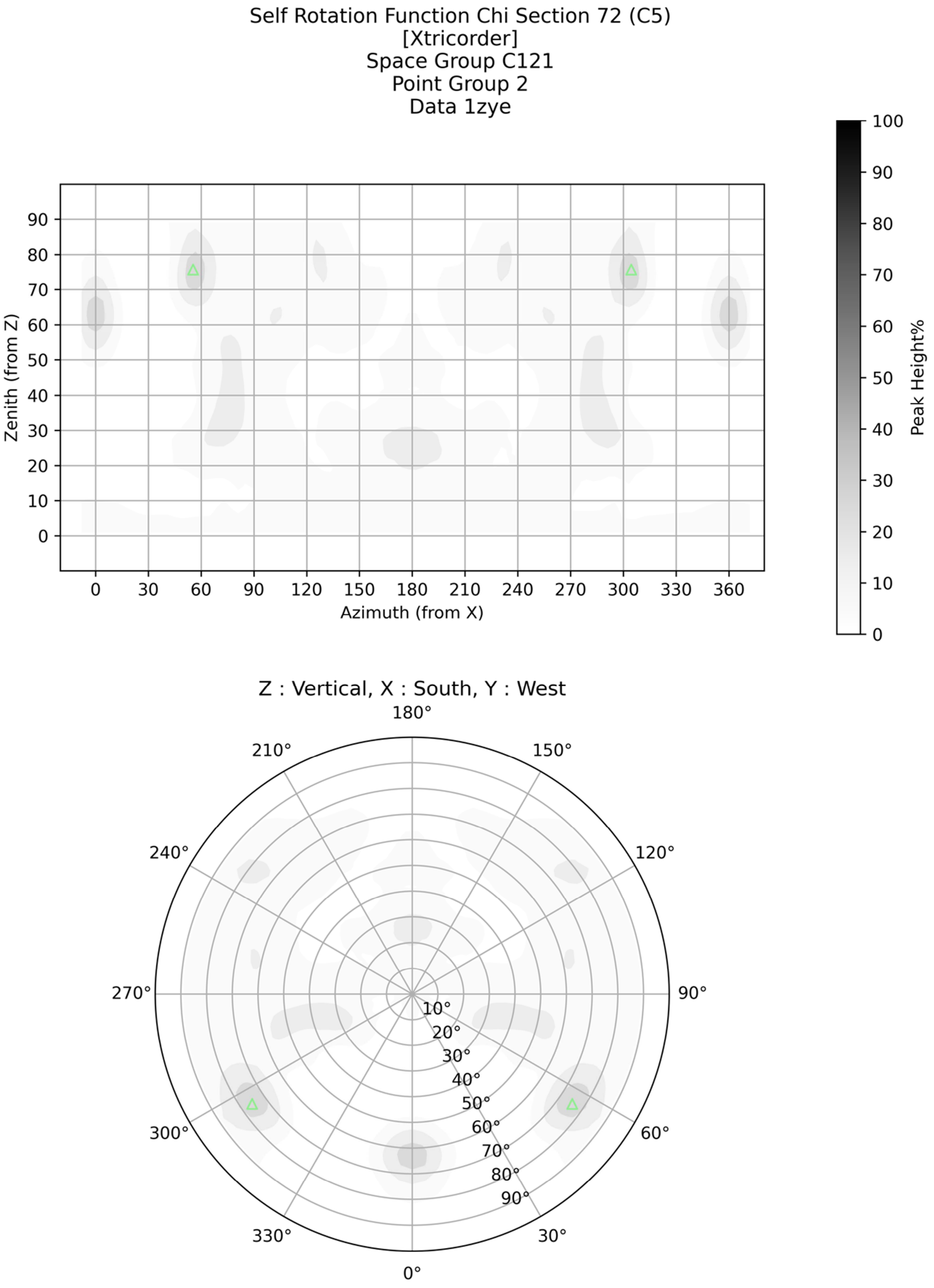
Self Rotation Function Chi Section 72 (C5)
[Xtricorder]
Space Group C121
Point Group 2
Data 1zye
Zenith (from Z)
Azimuth (from X)
Peak Height%
Z : Vertical, X : South, Y : West

e)

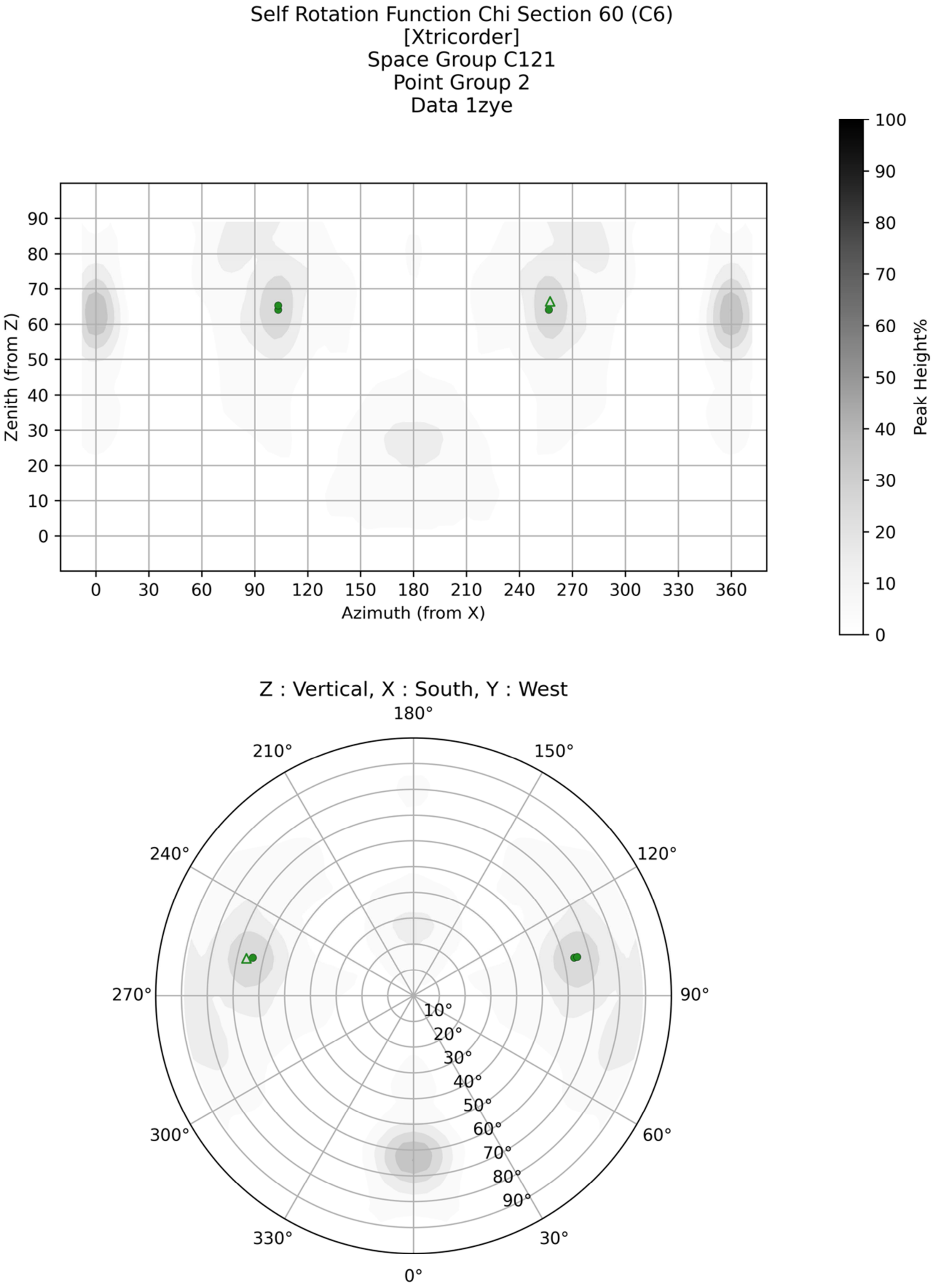
Self Rotation Function Chi Section 60 (C6)
[Xtricorder]
Space Group C121
Point Group 2
Data 1zye
Zenith (from Z)
Azimuth (from X)
Peak Height%
Z : Vertical, X : South, Y : West

f)

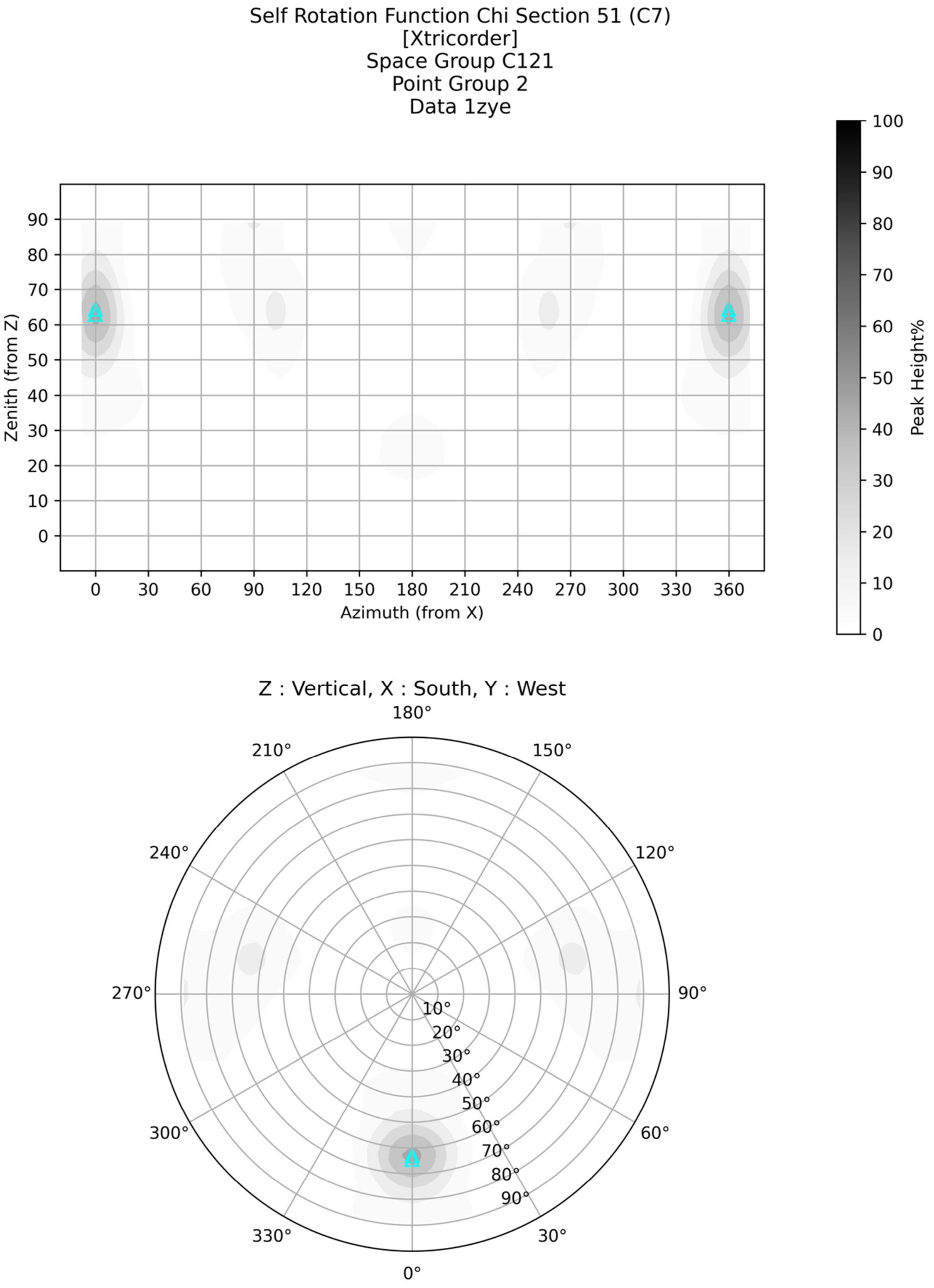
Self Rotation Function Chi Section 51 (C7)
[Xtricorder]
Space Group C121
Point Group 2
Data 1zye
Zenith (from Z)
Azimuth (from X)
Peak Height%
Z : Vertical, X : South, Y : West

g)

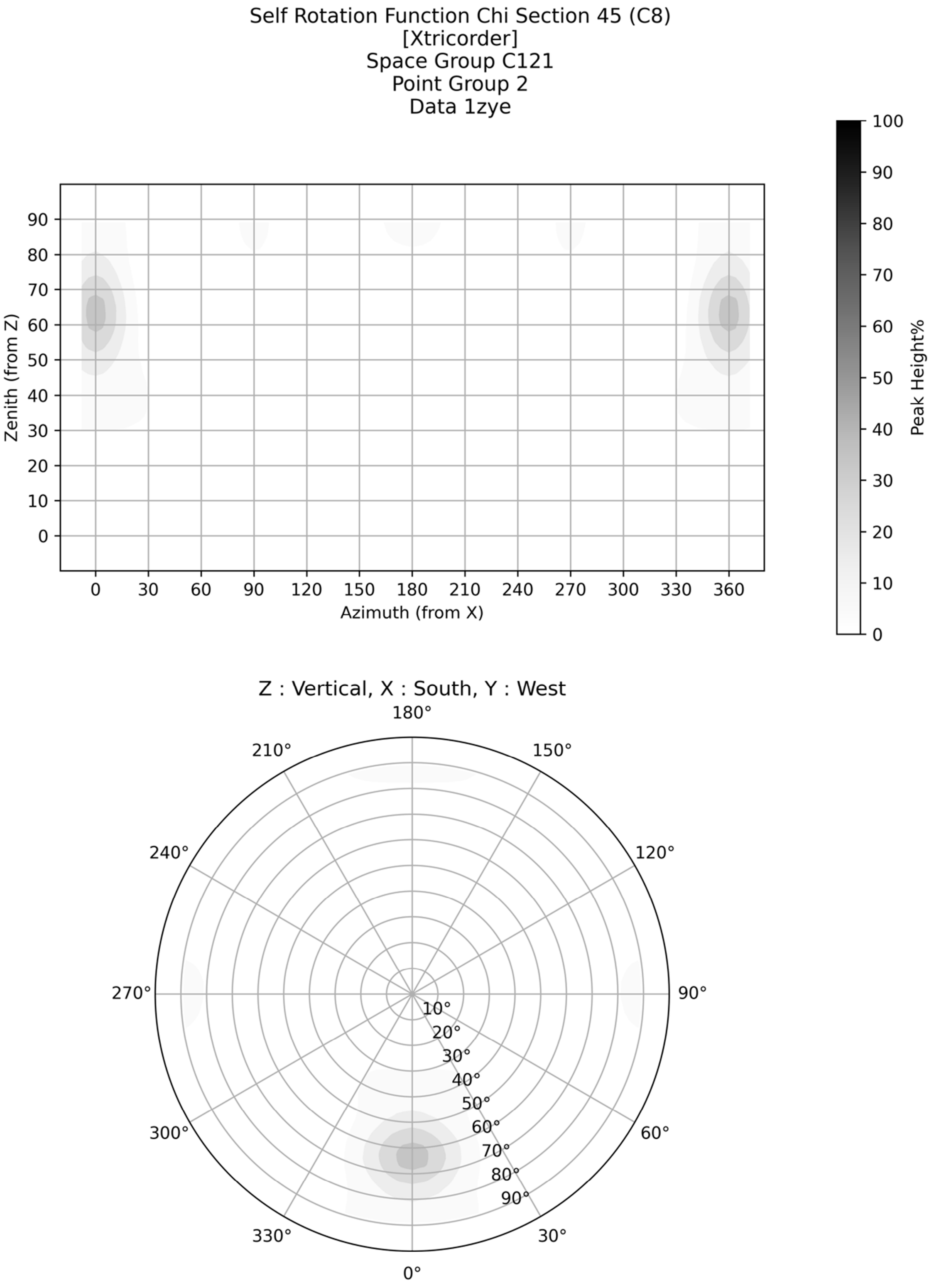
Self Rotation Function Chi Section 45 (C8)
[Xtricorder]
Space Group C121
Point Group 2
Data 1zye
Zenith (from Z)
Azimuth (from X)
Peak Height%
Z : Vertical, X : South, Y : West

h)

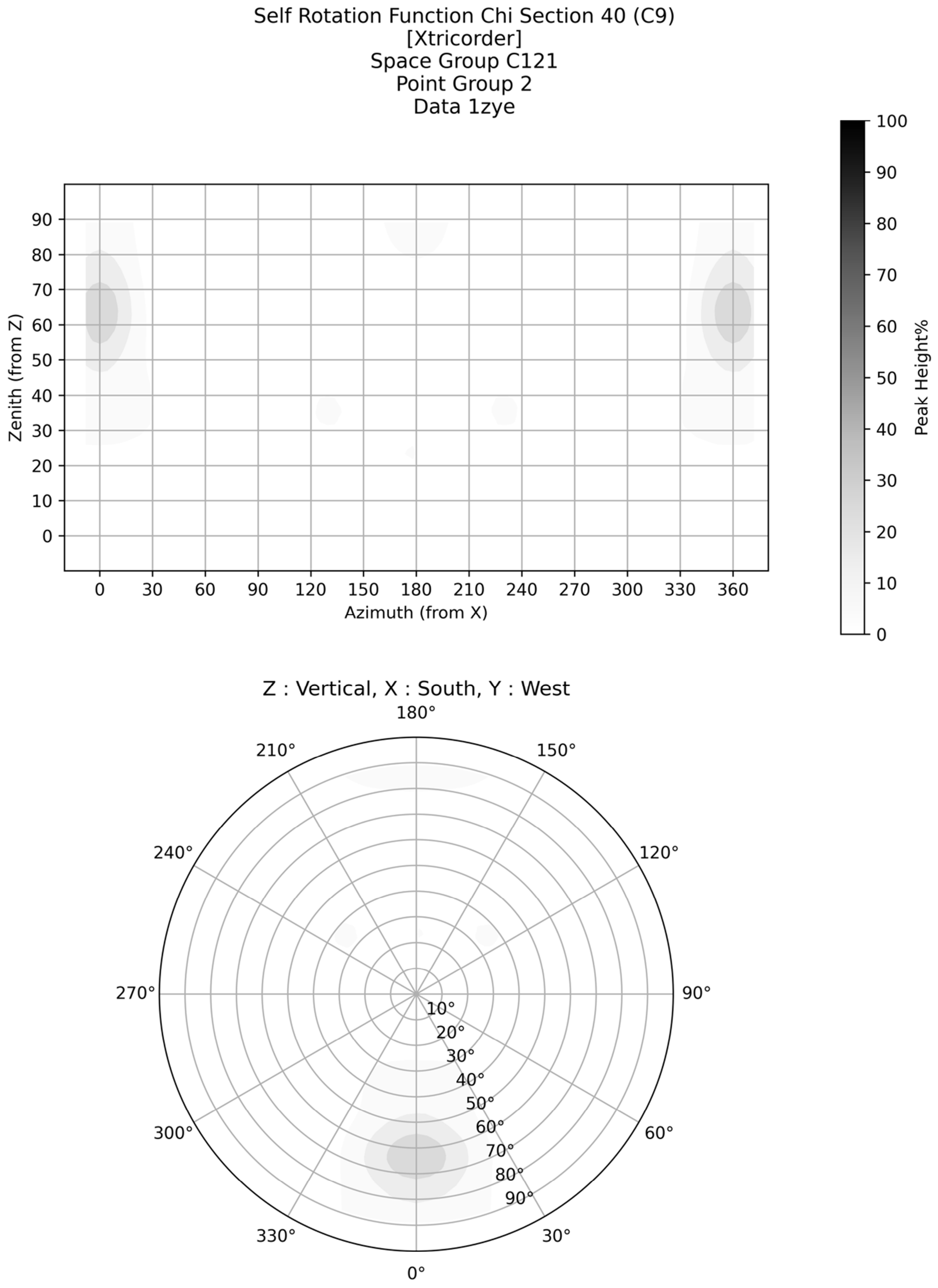
Self Rotation Function Chi Section 40 (C9)
[Xtricorder]
Space Group C121
Point Group 2
Data 1zye
Zenith (from Z)
Azimuth (from X)
Peak Height%
Z : Vertical, X : South, Y : West

Figure S4 Stacked SRF images for the protein structure 1ZYE used in machine learning. Images are shown at the resolution used for training and inference. a) Grid of 13 greyscale images, each representing a sequential slice from a single orientation stack used by the 3D convolutional neural network during both training and inference. MLE-Matthews Layer 1, 'Matthews-data', encodes Matthews probabilities by greyscale for each asymmetric unit content (range 1-36 by angular position around circumference) along with other relevant parameters: in the centre is a space group point group identifier; the strongest circular and dihedral, octahedral and icosahedral symmetries are encoded above the point group identifier (if present); and the order of the translational non-crystallographic symmetry is encoded below the point group identifier (if present). MLE-Matthews Layers 2-12 encode the information in the SRF composite-section diagrams, with axes and legends removed and other modifications for simplicity. Each Layer n for n in range 2-12 contains information about the corresponding C_n section: stars represent crystallographic symmetry, circles represent proper rotations within tolerances, and triangles represent rotations closest the section but outside the tolerances for proper rotations. Layer 13 encodes all higher order symmetries $n \geq 12$. b) The MLE-Matthews Control Layer image was used as a control during development; the image represents only the Matthews probabilities by greyscale.

a)

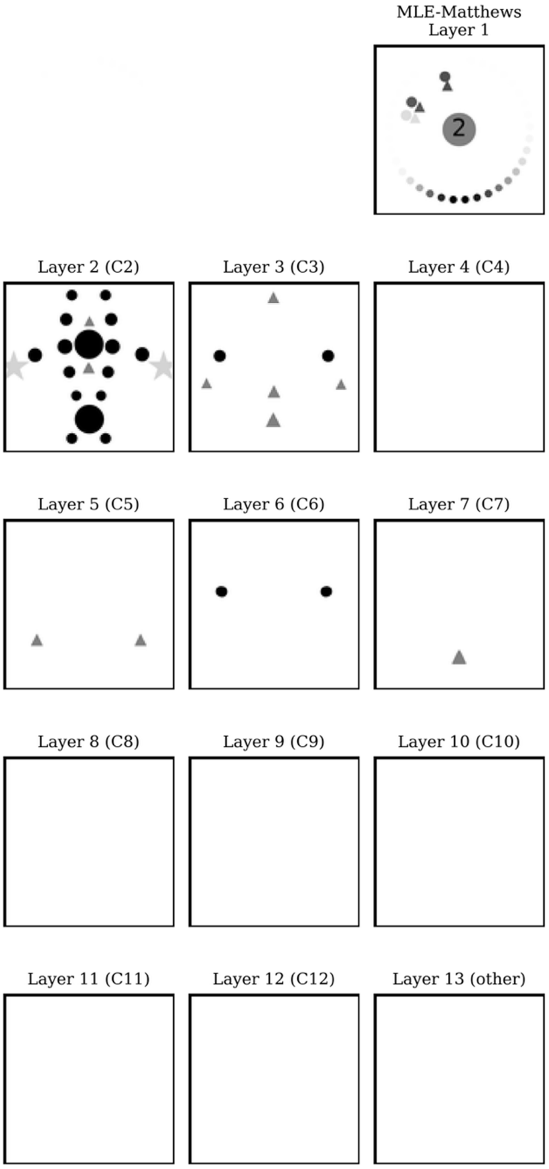
MLE-Matthews
Layer 1
2
Layer 2 (C2)
Layer 3 (C3)
Layer 4 (C4)
Layer 5 (C5)
Layer 6 (C6)
Layer 7 (C7)
Layer 8 (C8)
Layer 9 (C9)
Layer 10 (C10)
Layer 11 (C11)
Layer 12 (C12)
Layer 13 (other)

b)

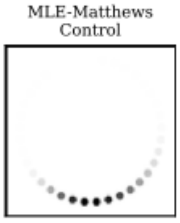
MLE-Matthews
Control

Figure S5 Distribution of structural and crystallographic properties across the dataset. Nine histograms summarizing the frequency of key parameters for structures and their crystallographic data. (a) Crystallographic point group. (b) Solvent content of the crystal lattice. (c) Data anisotropy, represented by ΔB (difference in B-factors along principal axes). (d) Assembly radius, defined as distance from centre of mass containing 90% of atoms. (e) Sphericity of assemblies, defined as the ratio of the longest principal axis to the shortest. (f) Twinning status of the crystallographic data. (g) Number of assemblies per asymmetric unit. (h) Number of residues per chain. (i) Number of chains per assembly. Each subplot uses logarithmic scaling on the y-axis. Histogram bins and x-axis labels are customized per feature.

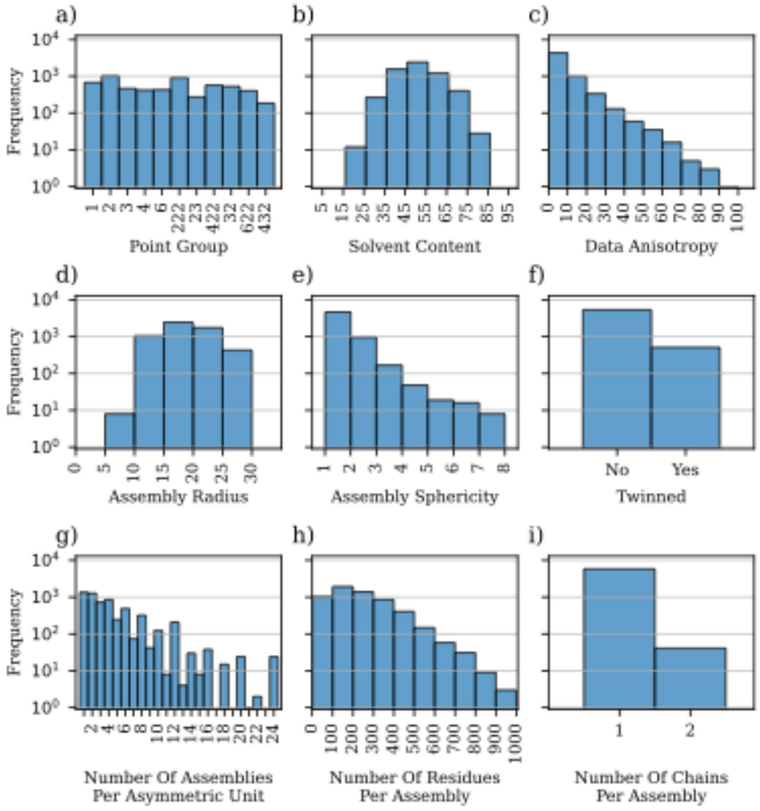

Figure S6 Relationship between symmetry and the number of assemblies per asymmetric unit in dataset. Stacked bar plots showing the distribution of symmetry orders (Cn and Dn) and number of assemblies per asymmetric unit for structures (a, b) For each symmetry order (n in Cn or Dn), the distribution of number of assemblies per asymmetric unit is shown, normalized across all symmetry types. Only n values from 2 to 12 are shown but the proportion of higher order $n \geq 13$ values are shown in white. (c, d) For each number of assemblies per asymmetric unit (ranging from 1 to 24), the frequency of associated symmetry orders (n in Cn or Dn) is shown, normalized across all bins. Colours correspond to symmetry order or number of assemblies per asymmetric unit value, as indicated in the legends. Frequencies are normalized within each number of assemblies per asymmetric unit bin (a, b) or symmetry order (c, d). Panel labels (a–d) correspond to each subplot.

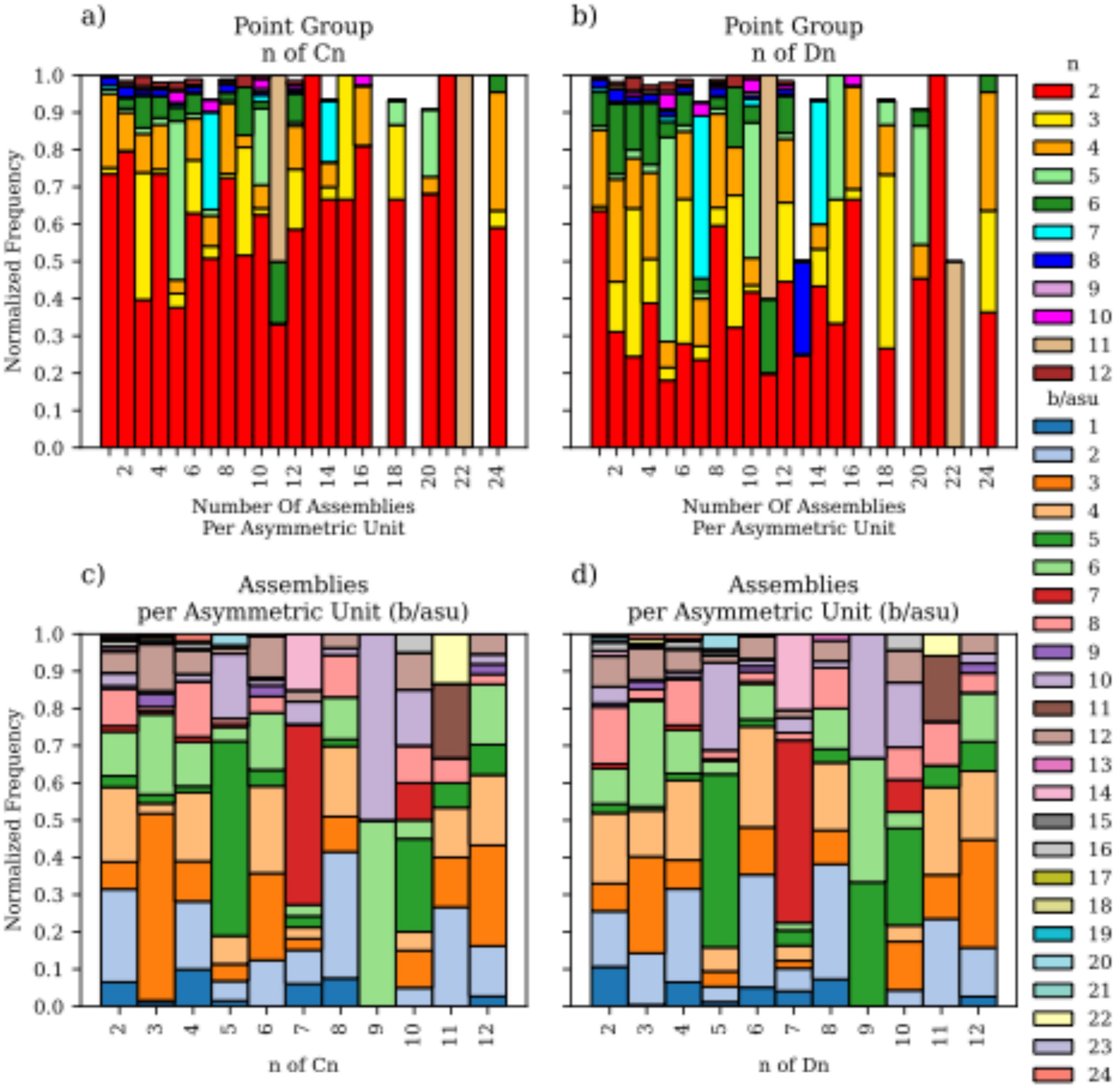
a)
Point Group
n of Cn
b)
Point Group
n of Dn
Normalized Frequency
Number Of Assemblies
Per Asymmetric Unit
c)
Assemblies
per Asymmetric Unit (b/asu)
d)
Assemblies
per Asymmetric Unit (b/asu)
n of Cn
n of Dn
n
b/asu
